# Restoration of Motor Function through Delayed Intraspinal Delivery of Human IL-10-Encoding Nucleoside-Modified mRNA after Spinal Cord Injury

**DOI:** 10.34133/research.0056

**Published:** 2023-03-09

**Authors:** László Gál, Tamás Bellák, Annamária Marton, Zoltán Fekécs, Drew Weissman, Dénes Török, Rachana Biju, Csaba Vizler, Rebeka Kristóf, Mitchell B. Beattie, Paulo J.C. Lin, Norbert Pardi, Antal Nógrádi, Krisztián Pajer

**Affiliations:** ^1^ Department of Anatomy, Histology and Embryology, Albert Szent-Györgyi Medical School, University of Szeged, Szeged, Hungary.; ^2^ National Biotechnology Laboratory, Institute of Genetics, Biological Research Centre, Szeged, Hungary.; ^3^ Institute of Biochemistry, Biological Research Centre, Szeged, Hungary.; ^4^ Department of Medicine, University of Pennsylvania, Philadelphia, PA, 19104, USA.; ^5^ Acuitas Therapeutics, Vancouver, BC, V6T 1Z3, Canada.

## Abstract

Efficient in vivo delivery of anti-inflammatory proteins to modulate the microenvironment of an injured spinal cord and promote neuroprotection and functional recovery is a great challenge. Nucleoside-modified messenger RNA (mRNA) has become a promising new modality that can be utilized for the safe and efficient delivery of therapeutic proteins. Here, we used lipid nanoparticle (LNP)-encapsulated human interleukin-10 (hIL-10)-encoding nucleoside-modified mRNA to induce neuroprotection and functional recovery following rat spinal cord contusion injury. Intralesional administration of hIL-10 mRNA-LNP to rats led to a remarkable reduction of the microglia/macrophage reaction in the injured spinal segment and induced significant functional recovery compared to controls. Furthermore, hIL-10 mRNA treatment induced increased expression in tissue inhibitor of matrix metalloproteinase 1 and ciliary neurotrophic factor levels in the affected spinal segment indicating a time-delayed secondary effect of IL-10 5 d after injection. Our results suggest that treatment with nucleoside-modified mRNAs encoding neuroprotective factors is an effective strategy for spinal cord injury repair.

## Introduction

Traumatic spinal cord injury (SCI) affects nearly 1.4 million North Americans, many of whom are younger than 30 years old [1]. SCI is a devastating disorder leading to loss of both gray and white matter and disrupts the connection the brain and rostral spinal cord from the caudal parts of the cord [2]. After the primary injury, number of neurons and glia cells die within days in the lesion area resulting in permanent, incurable functional deficits. Many of these injuries affect the long ascending and descending tracts, thus separating the lower spinal cord segments from the higher motor and sensory centers [3]. After the primary physical injury, the number of process begins called secondary injury, the extent of which is always larger than that of the primary injury [1]. Due to this phase, second set of symptoms can be observed in the injured spinal cord such as apoptosis, glutamate excitotoxicity, disruption of the blood–brain barrier, demyelination, and reactive astrogliosis [4]. Taken together, these processes induce extensive scar formation, Wallerian degeneration, and cavity formation [2–4].

Administration of interleukin-10 (IL-10) protein has shown promise in the treatment/cure of SCI, but safe and efficient drug delivery to the injured spinal cord represents an elusive goal. Several studies have demonstrated that administration or induced expression of IL-10 protein following experimental spinal cord injury improved neuronal survival and a certain level of functional recovery [5,6].

However, the short half-life of IL-10 and its instability in circulation represents a major challenge, necessitating repeated or continuous administration. Several strategies have been developed for the reliable delivery of neurotrophic factors or cytokines to injured spinal cord, but these approaches are often invasive and associated with adverse effects [5,7,8]. These delivery approaches have included viral vectors, repeated intrathecal/intravenous injections, and the use of osmotic pumps [5,8,9]. Therefore, development of a safe and controllable tool for cytokine delivery where the combined action of the identified biomolecules elicit definite morphological and functional improvement is critically important.

Messenger RNA (mRNA)-based therapy has recently emerged as a safe and very efficient approach that has wide applicability ranging from vaccination through protein replacement to gene editing [10–14]. mRNA-based therapy has several conceptual advantages over protein or other nucleic acid-based approaches. These advantages include the lack of insertional mutagenesis, continued production of the required protein for days, and no need of nuclear entry for the mRNA molecules. The most advanced mRNA delivery platform utilizes lipid nanoparticle (LNP)-encapsulated nucleoside-modified mRNA. Modification of the mRNA is critical to reduce inflammatory responses after mRNA delivery and increase protein production from mRNA [15,16]. LNP serves as an efficient carrier molecule for in vivo mRNA delivery, protecting the mRNA from rapid degradation and facilitating its cellular uptake. In this study, we utilized a human IL-10 (hIL-10)-encoding mRNA-LNP construct for the treatment of injured spinal cords in a rat model system.

The aim of this study was to investigate whether intraspinally applied nucleoside-modified hIL-10 mRNA-LNP was able to be transiently translated in the injured spinal cord and induce significant neuroprotection and locomotor recovery. Our results may open new avenues for mRNA-mediated gene transfer to improve the outcome of spinal cord injuries by precisely modulating protein expression by the host cells of the injured cord.

## Results

### Intraspinal delivery of enhanced green fluorescent protein mRNA-LNP results in transient protein production in the intact spinal cord

**Fig. 1.**
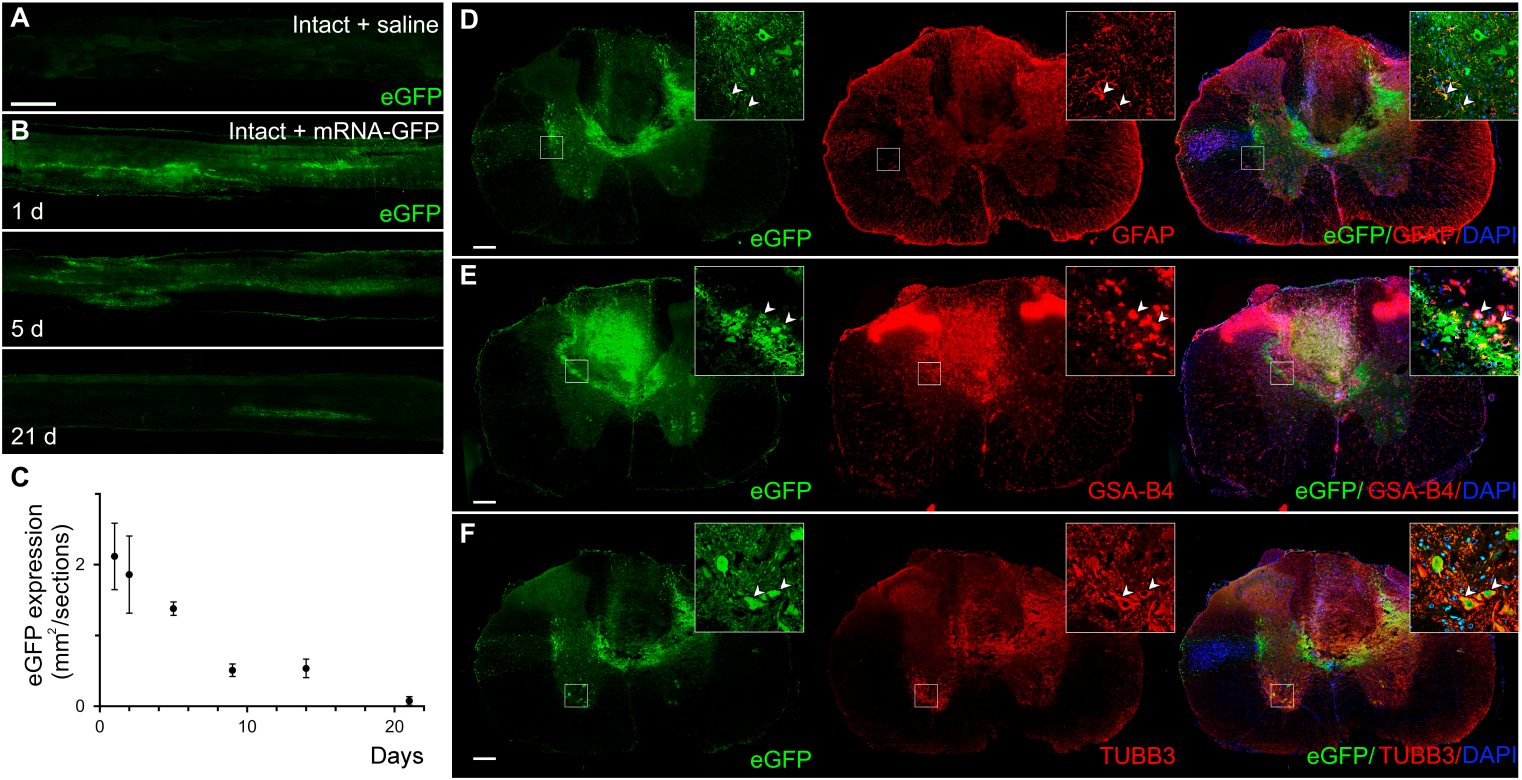
eGFP expression in intact rat spinal cords following intraspinal delivery of mRNA-LNP encoding eGFP. (A) No eGFP expression can be seen in the intact rat spinal cord without intraspinal administration of mRNA-LNP encoding eGFP. (B) eGFP expression in parasagittal sections of rat spinal cord 1, 5, and 21 d after the intraspinal injection of mRNA-LNP. The extent of eGFP-positive area is very limited 21 d after mRNA-LNP administration. (C) Quantification of the fluorescent eGFP signal after intraspinal injection of 3.0-μg mRNA-LNP. Note the marked drop in the size of eGFP+ area after 5 d. (D and E) Cross-sections of spinal cord show immunhistochemically detected eGFP in astrocytes (GFAP+) (D), in neurons (TUBB3+) (E), and in microglia/macrophages (GSA-B4+) (F) 1 d after the intraspinal mRNA-LNP administration. Data represent the mean ± SEM in (C) (*n* = 4; biologically independent experiments). Arrowheads show the colocalized cells. Scale bars in (A) = 1 mm and in (D) to (F) = 200 μm. DAPI, 4′,6-diamidino-2-phenylindole.

Recent findings from our laboratories showed that a single injection of low doses of LNP-formulated firefly luciferase-encoding mRNA (0.1 to 5 μg) resulted in high levels of protein production for up to 10 d depending on the dose and the site of delivery in mice [17]. To examine the duration and distribution of protein production from mRNA-LNP in the central nervous system (CNS), 3 μg of enhanced green fluorescent protein (eGFP) mRNA-LNP was pressure-injected into the intact rat spinal cord at the T10 vertebral level on day 0 (Fig. S1A). Strong immunofluorescent signal was observed rostrally and caudally from the injection site for 5 d after injection (Fig. 1A and B). The highest amount of eGFP expression was detected 1 d after injection and then a decreasing field of eGFP signal could be measured for up to 21 d (Fig. 1C), when GFP expression was restricted to a very small area around the injection site. The eGFP protein was mainly detected in glial fibrillary acidic protein (GFAP)-positive astrocytes and β-tubulin isotype 3A (TUBB3)-positive neurons in the affected segment, resulting in colocalization areas of eGFP/GFAP (22.5 ± 10.3%, standard error of the mean [SEM]) and eGFP/TUBB3 (29 ± 4.8%, SEM, Fig. 1D and F). As an extra cell population, GSA-B4-positive microglia/macrophages expressed eGFP along the injection channel only on day 1 after injection (Fig. 1E, eGFP/GSA-B4 colocalization area: 25 ± 7.5%, SEM). Iba-1, a microglia marker, also colocalized with eGFP in microglia cells (eGFP/Iba-1 colocalization area: 5.8 ± 1.3%, SEM, Fig. S3). eGFP was produced only by astrocytes and neurons 2 and 5 d after the mRNA-LNP administration (colocalization areas of eGFP/GFAP and eGFP/TUBB3 on day 2: 12.1 ± 4% and 19 ± 10.4%, SEM; on day 5: 29.7 ± 5.1% and 12.4 ± 1%, SEM; Fig. S2A to F). At later time points, eGFP expression of neurons ceased as it was maintained only in astrocytes up to day 21 (colocalization area of eGFP/GFAP on day 9: 18.6 ± 7.2%, SEM; on day 14: 15.3 ± 5.8%, SEM; and on day 21: 14.3 ± 6.3%, SEM; Fig. S2G to O).

#### Protein expression after delayed intralesional delivery of eGFP-mRNA-LNP in the injured spinal cord

**Fig. 2.**
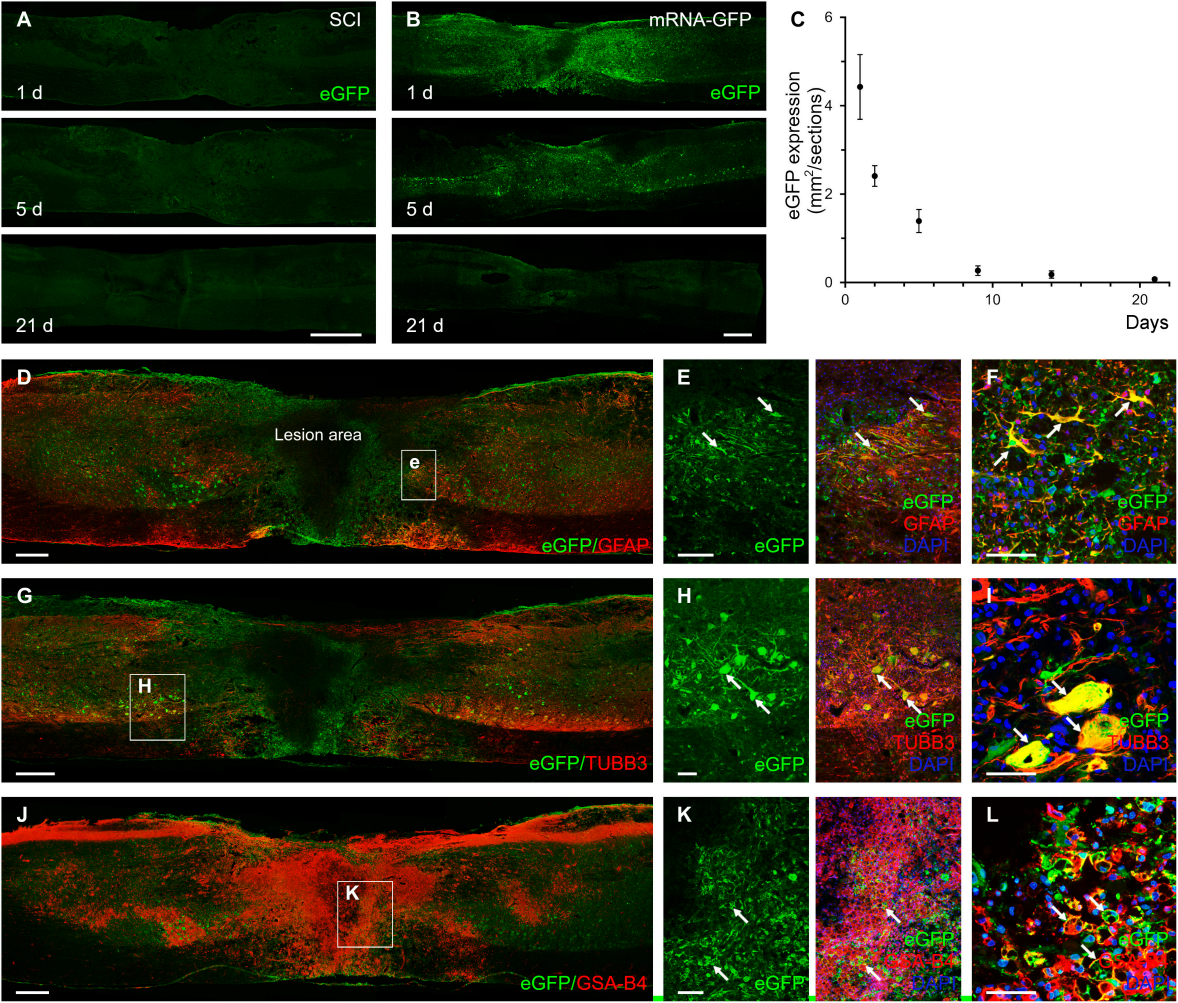
Protein production from eGFP mRNA-LNP in injured rat spinal cord after intralesional delivery. (A) No eGFP expression is seen in the untreated SCI group. SCI animals received spinal cord contusion injury and 3-μl saline 7 d after injury. (B) eGFP expression in injured spinal cords followed up to 21 d after intraspinal mRNA-LNP encoding eGFP administration. (C) Quantification of eGFP expression in parasagittal sections of spinal cords at various time points in the mRNA-GFP animals. The eGFP expression drops dramatically after 5 d. (D to L) Astrocytes (GFAP+) (D to F), neurons (TUBB3+) (G to I), and microglia/macrophages (GSA-B4+) (J to L) expressed eGFP 1 d after intraspinal delivery of mRNA-LNP encoding eGFP. (F, I, and L) Higher magnification clearly shows the presence of eGFP in the cytoplasm of astrocytes, neurons, and microglia/macrophages. Arrows show GFAP-, TUBB3-, and GSA-B4-positive cells colocalizing eGFP. Data represent the mean ± SEM in (C) (*n* = 4; biologically independent experiments). Scale bars in (A) and (B) = 1 mm, in (D), (G), and (J) = 500 μm, in (E), (H), and (K) = 100 μm, and in (F), (I), and (L) = 50 μm.

We next examined the expression kinetics of protein production from eGFP mRNA-LNP in injured spinal cords (mRNA-GFP group). Strong eGFP immunofluorescent signal was detected at the lesion site and rostrally and caudally from the end of the lesion cavity following intralesional delivery of mRNA-LNP encoding eGFP (Fig. 2B). The greatest area of eGFP expression was measured at 1 d after the injection of mRNA-LNP followed by a slow decrease in protein production up to 21 d (Fig. 2B). To determine the cell types transfected by eGFP mRNA-LNP, paramedian sagittal spinal cord tissue sections were mapped for eGFP colabeling with GFAP (astrocytes), TUBB3 (neurons), GSA-B4 (microglia/macrophages), and Iba-1 (microglia). Up to day 5 after injection eGFP/GFAP-positive astrocytes, eGFP/GSA-B4-positive microglia/macrophages, and eGFP/Iba-1-positive microglia cells were detected mainly around the lesion site (colocalization area of eGFP/GFAP on day 1: 41.6 ± 13.4%, SEM; colocalization area of eGFP/GSA-B4 on day 1: 42.9 ± 3.6%, SEM; colocalization area of eGFP/Iba-1 on day 1: 22.8 ± 4.1%, SEM; colocalization area of eGFP/GFAP on day 2: 42.1 ± 1.8%, SEM; colocalization area of eGFP/GSA-B4 on day 2: 26.9 ± 3.8%, SEM; colocalization area of eGFP/Iba-1 on day 2: 27.8 ± 6.5%, SEM; colocalization area of eGFP/GFAP on day 5: 32.7 ± 2.2%, SEM; colocalization area of eGFP/GSA-B4 on day 5: 12 ± 1.8%, SEM; colocalization area of eGFP/Iba-1 on day 5: 15.2 ± 3.3%, SEM; Fig. 2D to F and J to L and Figs. S4A and B and S5A to C) while rostrally and caudally from the lesion, and a number of TUBB3-positive neurons and their processes expressed strongly eGFP (colocalization area of eGFP/TUBB3 on day 1: 40.5 ± 2.5%, SEM; colocalization area of eGFP/TUBB3 on day 2: 32.3 ± 12.9% SEM; colocalization area of eGFP/TUBB3 on day 5: 27.6 ± 5.7%, SEM; Fig. 2G to I and Fig. S4A and B). On days 9 and 14 after mRNA-LNP injection, only neurons and astrocytes showed eGFP expression (colocalization areas of eGFP/GFAP on days 9 and 14: 31.7 ± 5.2%, SEM and 20.3 ± 3.7%, SEM; colocalization area of eGFP/TUBB3 on days 9 and 14: 16.7 ± 3.6%, SEM and 8.3 ± 4.8%. SEM; Figs. S4C to E and S5D to F), while on day 21 exclusively few astrocytes showed colocalization with the eGFP protein (colocalization area of eGFP/GFAP on day 21: 11.8 ± 4.4, SEM, Fig. S4F). These results clearly demonstrated that intralesional administration of mRNA-LNP into the injured spinal cord resulted in active and transient translation of mRNA to protein.

#### Expression of hIL-10 mRNA-LNP in the injured spinal cord: Kinetics and localization

**Fig. 3.**
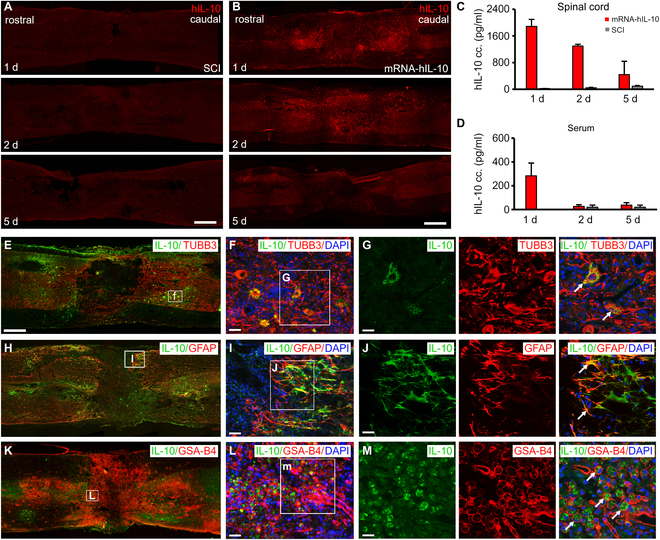
hIL-10 expression in injured spinal cords following intralesional delivery of mRNA-LNP encoding hIL-10. (A) No hIL-10 expression was detected in the injured rat spinal cord (SCI group). SCI animals received 3 μl of saline intralesionally 7 d after the spinal cord contusion injury. (B) hIL-10 expression detected in paramedian sagittal sections of injured rat spinal cords 1, 2, and 5 d after intraspinal delivery of mRNA-LNP encoding hIL-10. (C) and (D) Production of hIL-10 in injured spinal cord and serum after hIL-10 mRNA-LNP delivery determined by ELISA. (E to G) Rat neurons express hIL-10 1 d after intralesional mRNA-LNP administration. (H to J) Rat astrocytes produced hIL-10 in the close vicinity of the lesion 1 d after mRNA-LNP delivery. (K to M) GSA-B4-positive cells colocalized with hIL-10 in the lesion area 1 d after mRNA-LNP delivery. Data represent the mean ± SEM; (C and D) *n* = 4, biologically independent experiments. Arrows show the colocalized cells. Scale bar in (A) and (B) = 800 μm, in (E) = 750 μm, in (F) and (L) = 30 μm, in (I) 25 = μm, and in (G), (J), and (M) = 20 μm.

Next, the expression and synthesis of the anti-inflammatory hIL-10 was tested after delayed intralesional delivery of 3 μg of hIL-10 mRNA-LNP following spinal cord contusion injury in rats (mRNA-hIL-10 group). Paramedian sagittal sections of the spinal cords were immunostained with hIL-10-specific antibody, and strong immunofluorescence was detected rostrally and caudally from the injection site on days 1 and 2 and weak expression 5 d after the injection of hIL-10 mRNA-LNP (Fig. 3A and B). Enzyme-linked immunosorbent assay (ELISA) results supported the immunohistochemical findings as high hIL-10 expression was detected on day 1 after the injection followed by a steady decline until day 5 in the injured spinal cord (Fig. 3C). Interestingly, hIL-10 was also detected in the blood serum 1 d after intralesional mRNA treatment, but at later time points, only insignificant amounts of hIL-10 was detectable (Fig. 3D).

On day 1 after mRNA-LNP injection, granular expression of hIL-10 could be observed in the cell body but not in the processes of TUBB3-positive neurons in the affected segment (colocalization area of hIL-10/TUBB3: 7.5 ± 1.6%, SEM; Fig. 3E to G).

hIL-10 was also found to be expressed in the astrocytes in the perilesional area (colocalization area of hIL-10/GFAP: 10.7 ± 2.8%, SEM; Fig. 3H to J), while a number of GSA-B4-positive microglia/macrophages contained hIL-10-positive profiles at the interface between the lesion and preserved spinal cord (colocalization area of hIL-10/GSA-B4: 2.5 ± 0.8%, SEM). Iba-1 also colocalizes in microglia with hIL-10 (colocalization area of hIL-10/Iba-1: 11.3 ± 5.8%, SEM; Fig. 3K to M and Fig. S6). A similar expression pattern of hIL-10 was noticed on day 2 after the mRNA delivery (colocalization areas of hIL-10/GFAP and hIL-10/TUBB3: 7.6 ± 3.2%, SEM and 4.7 ± 1.3%, SEM; Fig. S7A). Five days after mRNA-LNP administration, hIL-10 protein expression was observed only in the soma of neurons and astrocytes in the close vicinity of the lesion (colocalization areas of hIL-10/GFAP and hIL-10/TUBB3: 5.8 ± 1.9%, SEM and 2.9 ± 2.7%, SEM, respectively; Fig. S7B). In contrast to the translation of eGFP mRNA-LNP in the injured spinal cord, hIL-10 expression was not detected 9 or 14 d after intralesional administration. These results suggest that the host cells of the spinal cord were transfected by the hIL-10 mRNA-LNP leading to active protein synthesis at least for 5 d, while the activated microglia/macrophages likely phagocytosed the already transfected cells.

In the case when osmotic pumps were used (osm-hIL-10 group), it was not possible to reliably determine the tissue level of IL-10 in the spinal cord or the further fate of delivered IL-10 in the spinal cord.

#### Improved locomotor pattern after hIL-10 protein treatment via osmotic pump or mRNA-LNP delivery

**Fig. 4.**
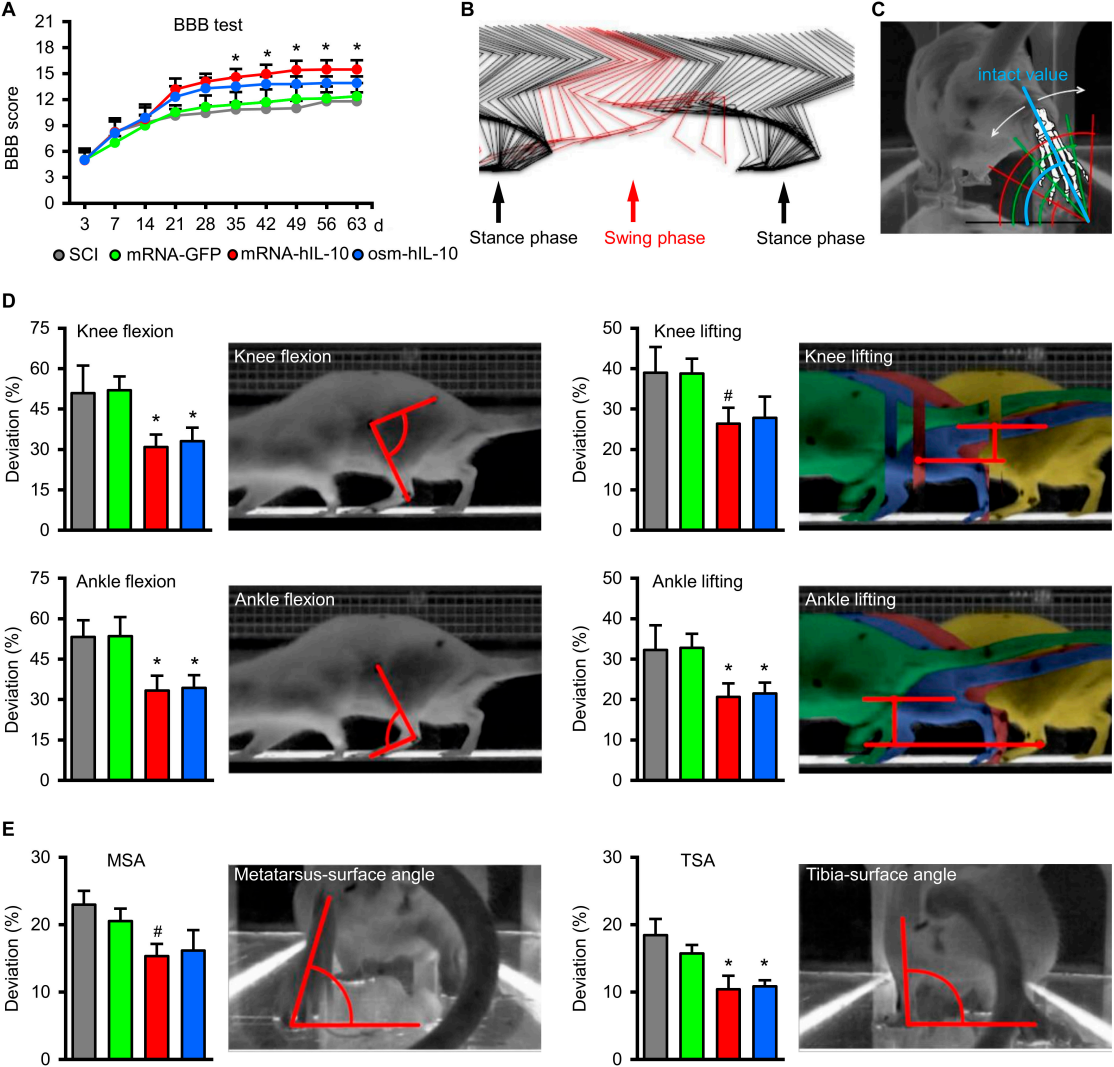
Delayed intraspinal administration of mRNA-LNP encoding hIL-10 improves locomotor function. (A) Open-field locomotor test (BBB) shows significant improvement of hIL-10-treated animals (mRNA-hIL-10 and osm-hIL-10 group) compared with their controls. Asterisks indicate significant difference between the hIL-10-treated animals (mRNA-hIL-10 and osm-hIL-10 group) and SCI and mRNA-GFP groups at various time points. (B) The image shows every position of the measured bones during 1 intact step cycle from the lateral aspect. The step cycle can be divided into stance phase (black) and swing phase (red). (C) The measurement of the rear-view parameters is based on the angle enclosed by a selected bone and the floor plate. The intact value is displayed in blue, while green and red angles represent the deviations followed by contusion injury, respectively. White arrows show the deviations in both directions. (D and E) Kinematic analysis of the animals in the various groups 9 weeks after injury. Note the significantly improved parameters of the hIL-10-treated animals (mRNA-hIL-10 and osm-hIL-10 group) compared with SCI and mRNA-GFP groups. Data represent the mean ± SEM. (A, D, and E) *n* = 8, biologically independent experiments. *P < 0.05 and indicates significant difference among SCI, mRNA-GFP vs. mRNA-hIL-10 and osm-hIL-10 groups. #*P* < 0.05 and shows significant difference between SCI, mRNA-GFP vs. mRNA-IL10 group. Data were analyzed using the 2-way ANOVA (A) or 1-way ANOVA with LSD multiple comparisons tests (D and E).

To test the locomotor improvement after hIL-10 treatment through either the use of an osmotic pump to deliver the recombinant hIL-10 protein (osm-hIL-10) or by hIL-10 mRNA-LNP administration (mRNA-hIL-10), we used the Basso, Beattie, Bresnahan (BBB) locomotor rating scale and the kinematic analysis developed in our laboratory [18,19]. From week 3 after injury, a notable improvement based on the BBB score was observed both in the mRNA-hIL-10 and osm-hIL-10 animals followed by a continuous motor improvement up to 9 weeks (Fig. 4A). In the osm-hIL-10 group, animals showed similar locomotor pattern as mRNA-hIL-10 treatment group, although mRNA-hIL-10 animals produced slightly, but nonsignificantly better BBB values than the animals that received IL-10 treatment via osmotic pumps. Statistically significant differences were found between the hIL-10-treated animals (mRNA-hIL-10 and osm-hIL-10 groups) and their controls (SCI and mRNA-GFP groups; *P* ≤ 0.05; Fig 4A).

The kinematics analysis of IL-10-treated (mRNA-hIL-10 and osm-hIL-10 groups) and control rats (SCI and mRNA-GFP groups) was performed to provide quantitative information about knee flexion and lifting, ankle flexion and lifting, metatarsus surface angle, and tibia surface angle at 9 weeks after the injury (Fig. 4B to D). Consistent with the BBB results (9 weeks after injury), the kinematic analysis revealed that hIL-10-treated animals (mRNA-hIL-10 and osm-hIL-10 group) displayed a significant improvement in all examined parameters compared to control animals (SCI and mRNA-GFP groups) that displayed only slight recovery after SCI.

#### Morphological restoration following intralesional delivery of mRNA-LNP encoding hIL-10

**Fig. 5.**
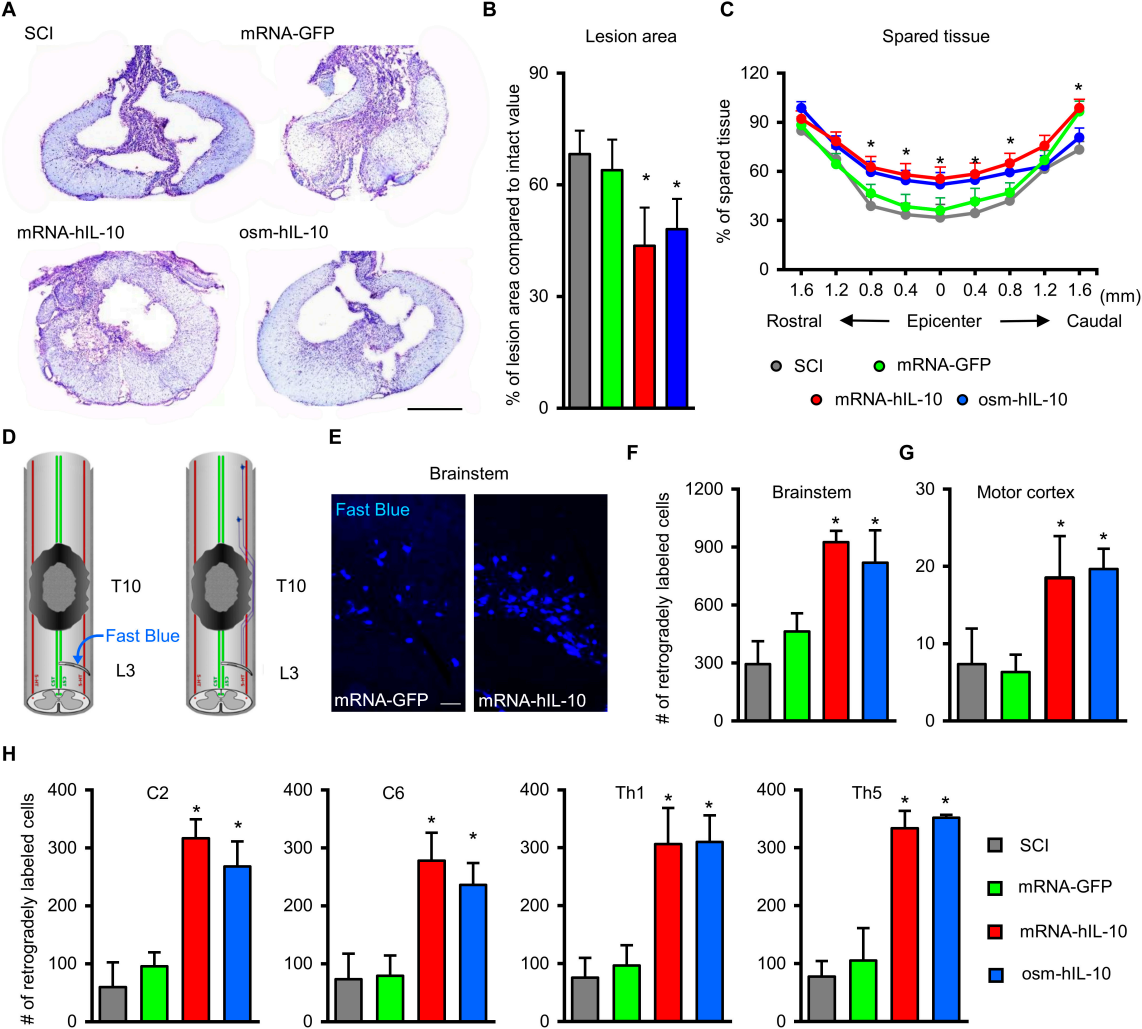
Delayed intraspinal administration of mRNA-LNP encoding hIL-10 induces tissue sparing. (A) Representative images of cresyl-violet-stained sections taken at 100 μm rostrally from the lesion epicenter. (B) Quantification of lesion area shows that hIL-10 treatment resulted in significantly reduced size of injury following SCI. (C) Improved tissue sparing can be seen rostral and caudal to lesion epicenter in hIL-10-treated groups (mRNA-hIL-10 and osm-hIL-10) compared with SCI and mRNA-GFP control animals. (D) Schematic image shows the retrograde labeling procedure. FB crystals were placed into the right hemisection gap of the L3 spinal segment. (E) Retrogradely labeled neurons are shown in the brainstem of mRNA-GFP and mRNA-IL10 animals. (F to H) Quantification of retrogradely labeled neurons in the brainstem (F), in the motor cortex (G) and in various spinal segments rostrally from the contusion injury (H). Data represent the mean ± SEM. (A, B, C, F, G, and H) *n* = 4, biologically independent experiments. *P < 0.05 and indicates significant difference between SCI, mRNA-GFP vs. mRNA-hIL-10, and osm-hIL-10 groups. Data were analyzed using 1-way ANOVA with LSD multiple comparisons test (A, B, C, F, G, and H). Scale bar in (A) = 500 μm and in (E) = 100 μm.

Morphometric analysis of the lesion area at the epicenter and spared tissue was performed 9 weeks after the injury. Within the lesion area, a rostro-caudally extended cavity was observed with some cellular debris in SCI groups (Fig. 5A). Administration of hIL-10 (mRNA-hIL-10 and osm-hIL-10) resulted in significantly smaller lesion area at the epicenter of the injury and significantly enhanced tissue sparing (Fig. 5B and C).

Next, we evaluated whether hIL-10 treatment preserved the connections between the segments caudal to the lesion and various cranial parts of the CNS. To study proprio- and supraspinal connections of the injured spinal cord, few crystals of the retrograde tracer Fast Blue (FB) were placed caudally to the injury into the right L3 segment and the retrogradely labeled neuronal somata in the spinal cord, brainstem, and somatomotor cortex were mapped (Fig. 5D and E). Significantly higher numbers of FB-labeled supraspinal (brainstem and motor cortex) and propriospinal (Th5, Th1, C6, and C2 spinal segments) neurons were found in the animals treated with hIL-10 (both mRNA-hIL-10 and osm-hIL-10 groups) than in their controls (SCI and mRNA-GFP groups; Fig. 5F to H). The number of retrogradely labeled neurons in C2 and C6 spinal segments of the mRNA-hIL-10-treated animals was somewhat higher than in the osm-hIL-10 group, but this difference did not achieve statistical significance.

#### mRNA-LNP-induced hIL-10 treatment decreases the microglia/macrophage reaction and modulates cytokine expression in the injured spinal cord

**Fig. 6.**
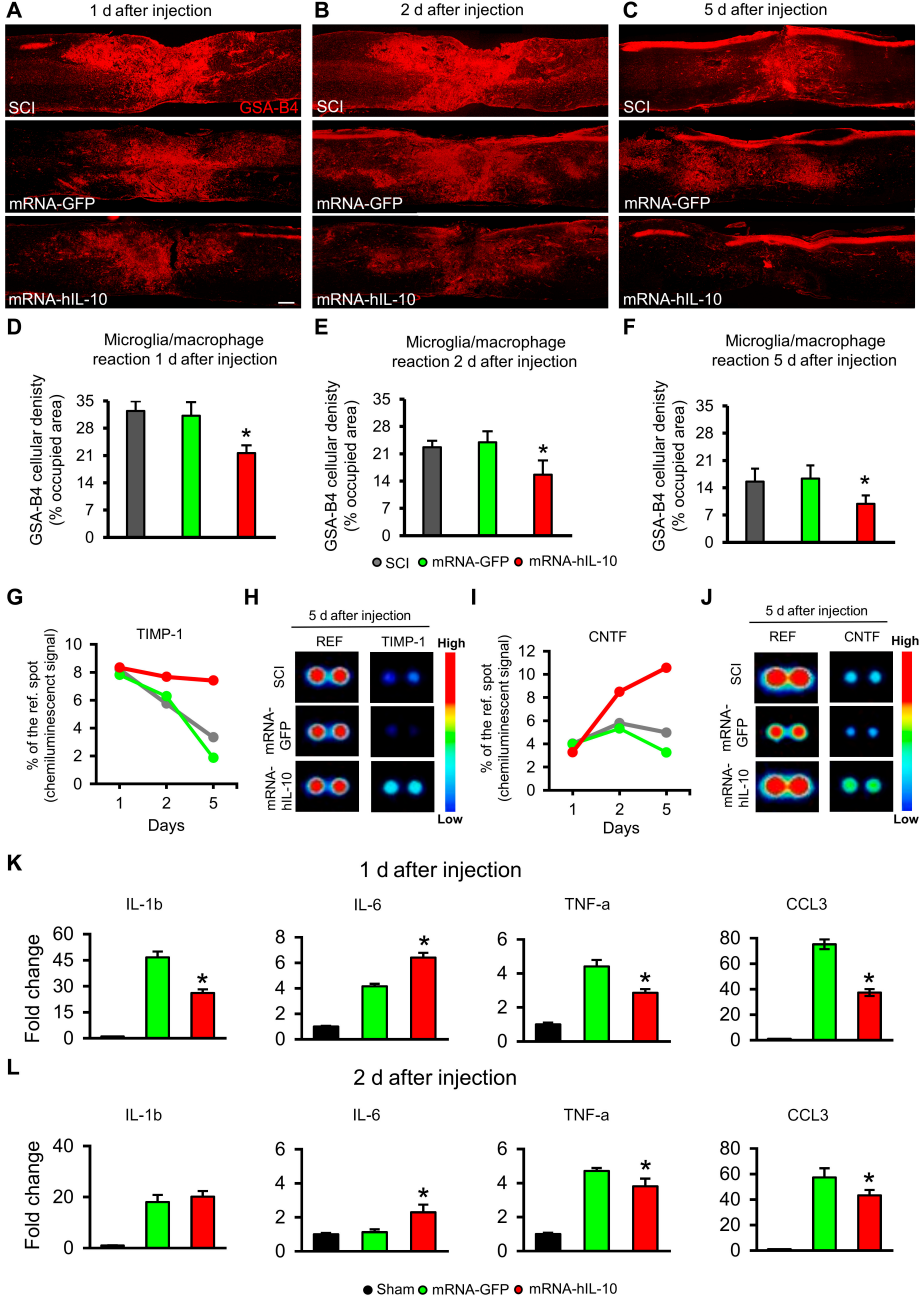
Microglia/macrophage and cytokine changes after mRNA LNP encoding hIL-10 treatment in the injured spinal cord. (A to C) Representative images of paramedian sagittal spinal cord sections show the GSA-B4 reactivity 1, 2, and 5 d after intralesional delivery of saline (A), mRNA-LNP encoding eGFP (B), and mRNA-LNP encoding hIL-10 (C) in the lesion area. (D to F) Quantification of microglia/macrophage (GSA-B4) density in the sagittal sections of the spinal cord revealed significantly decreased level of GSA-B4 at all examined time points in the hIL-10 mRNA-treated group (mRNA-hIL-10) compared with the SCI and mRNA-GFP groups. (G to J) Rodent cytokine level changes were assessed by using the Proteome Profiler array (ARY008) in the spinal cords of SCI, mRNA-GFP, and mRNA-hIL-10 groups, comparing the relative levels of 29 rat cytokines. The chemiluminescence signal of spots were compared with reference spots and expressed as % of those. Higher chemiluminescence signals of tissue inhibitor of matrix metalloproteinase 1 (TIMP-1) (G) and ciliary neurotrophic factor (CNTF) (I) were detected in the mRNA-hIL-10 group compared with the SCI and mRNA-GFP groups 2 and 5 d after the mRNA-LNP treatment. (H) and (J) show representative images of reference control, TIMP-1, and CNTF spots 5 d after the saline or mRNA-LNP injection. (K to L) Rat cytokine changes 1 and 2 d after mRNA-LNP administration detected by PCR analysis. Quantification of IL-6 mRNA in the spinal cord showed significantly increased levels, while that of TNF-a and CCL3 mRNAs revealed significantly decreased levels at both examined time points in the hIL-10 mRNA-treated group (mRNA-hIL-10) compared with the mRNA-GFP group. IL1-b mRNA levels were significantly decreased on day 1 but showed nonsignificant changes on day 2 after treatment in the hIL-10 mRNA-treated animals. Data were analyzed by using 1-way ANOVA with LSD multiple comparisons test. Data represent the mean ± SEM. (D to F) *n* = 4; (K and L) *n* = 3 each group, biologically independent experiments. (D to F) *P < 0.05 indicates significant difference between SCI and mRNA-GFP vs. mRNA-hIL-10. (K and L) * indicates statistically significant difference (*P* < 0.05) between mRNA-GFP vs. mRNA-hIL-10 groups. Scale bar in (A) = 200 μm.

It is well known that IL-10 is able to suppress inflammatory cytokine expression and the activation of inflammatory macrophages in injured spinal cord [20]. In the next series of experiments, we investigated whether the intralesional administration of hIL-10 mRNA-LNP alters the morphological and thus the molecular microenvironment of the lesion area. First, we examined and quantified the GSA-B4-positive microglia/macrophage densities 1, 2, and 5 d after injection in the affected spinal segment. Strong GSA-B4 densities were detected in SCI animals, whose spinal cord displayed high GSA-B4 expression in the epicenter of lesion with considerably weaker staining intensities detected rostrally and caudally from the lesion cavity. Similar expression of CD68 and Iba-1 was observed (Figs. S8A to C and S9A to C). Similar changes were found in the animals receiving eGFP mRNA-LNP at all examined time points (Fig. 6A and B). In contrast, moderately decreased GSA-B4 densities were observed following intralesional delivery of hIL-10 mRNA-LNP (Fig. 6C). The quantitative analysis of CD68, Iba-1, and GSA-B4 densities clearly showed that intralesional administration of hIL-10 mRNA-LNP significantly decreased the densities of CD68-, Iba-1-, and GSA-B4-positive cells compared with the injured spinal cords of SCI and mRNA-GFP animals at all examined time points (Fig. 6D to F and Figs. S8D to F and S9D to F). The significantly suppressed level of microglia/macrophage densities found in the mRNA-hIL-10 group moved us to hypothesize that there could be a differential production of cytokines in the control (SCI and mRNA-GFP groups) and treated (mRNA-hIL-10 group) spinal cords. We evaluated semiquantitatively the expression of 29 cytokines through the use of the Proteome Profiler array and determined the expression in pooled samples (Fig. 6G to J). On day 5 after hIL-10-LNP treatment, remarkably higher chemiluminescence signal of tissue inhibitor of matrix metalloproteinase 1 (TIMP-1) and ciliary neurotrophic factor (CNTF) was measured in the affected segment of the mRNA-hIL-10 group compared with the SCI and the mRNA-GFP animals. Although protein expression of hIL-10 was markedly decreased 5 d after intraspinal administration of hIL-10 mRNA (Fig. 3C), slightly increased expression of TIMP-1 and CNTF appeared as a time-delayed secondary effect. The circulating cytokines were also evaluated within the first 5 d in blood serum following intralesional injection of saline (SCI animals) or mRNA-LNP (mRNA-GFP and mRNA-hIL-10 groups) into the injured cords. No considerable change of cytokine levels was observed in the blood serum of the mRNA-hIL-10 animals compared with that of the SCI or mRNA-GFP animals (Fig. S10), except for the mild decrease of serum RANTES levels in the mRNA-hIL-10 group compared with the SCI and the mRNA-GFP animals by day 5 after treatment. In contrast, cytokine-induced neutrophil chemoattractant-1 (CINC-1) serum levels were mildly depressed in mRNA-hIL-10 and mRNA-GFP animals on day 2 compared with the SCI animals. These results suggest that intralesional administration of mRNA-LNP encoding hIL-10 has strong anti-inflammatory effect and results in a delayed expression of potent neuromodulatory factors in the affected spinal segments [21]. Rat cytokine changes 1 and 2 d after mRNA-LNP administration were detected by polymerase chain reaction (PCR) analysis (Fig. 6K and L). Quantification of IL-6 mRNA in the spinal cord showed significantly increased levels, while that of tumor necrosis factor-a (TNF-a) and chemokine (C-C motif) ligand 3 (CCL3) mRNAs revealed significantly decreased levels of these cytokines at both examined time points in the hIL-10 mRNA-treated group (mRNA-hIL-10) compared with the mRNA-GFP group. IL1-b mRNA levels were significantly decreased on day 1 but showed nonsignificant changes on day 2 after treatment in the hIL-10 mRNA-treated animals.

## Discussion

Functional and morphological recovery in the injured spinal cord is limited as the considerable primary physical cell death is followed by the development of an unfavorable microenvironment around the damaged neurons. Thus, neurons that are not destroyed by the primary physical damage in the injured cord fail to recover and reestablish their primary connections. In our study, we used nucleoside-modified mRNA to provide a new delivery approach resulting in the expression of the therapeutic protein IL-10 in the injured spinal cord, thus leading to improved neuroprotection and functional outcome.

Nucleoside-modified mRNA-LNP coronavirus disease 2019 (COVID-19) vaccines proved to be safe and effective in humans, opening the way for new applications of the platform such as protein replacement therapy and gene editing [22,23]. Here, we provide evidence that this revolutionary platform can potentially be utilized to successfully treat spinal cord injury and thus to restore motor function. In this work, the expression kinetics of the eGFP mRNA-LNP provided evidence for active translation of mRNA in intact and injured spinal cords. A single injection of a low dose of eGFP mRNA-LNP into intact and injured rat spinal cords resulted in a 3-week-long translation by spinal cord astrocytes. In contrast, neurons in the injured cords expressed GFP for 14 d, whereas eGFP expression in intact spinal cord neurons was limited to a 5-d-long period. It can be argued that the injury itself may have adjusted the cellular metabolic machinery leading to longer eGFP protein expression in the injured neurons. Interestingly, microglia/macrophages were positive for eGFP only for short time; however, astrocytes were able to express eGFP up to 21 d after intraspinal delivery of mRNA-LNP.

Based on the above results, mRNA-LNP encoding hIL-10 was administrated intraspinally at the same dose 1 week after the injury. hIL-10 is known to possess a marked anti-inflammatory effect and impart neuroprotection [5,6,24]. Indeed, we could prove hIL-10 protein production in neurons and astrocytes for at least 5 d after delivery while microglia/macrophages were found to express hIL-10 for 2 d only. The expression of hIL-10 in astrocytes and neurons indicates that these cell types were primarily targeted by the hIL-10 mRNA-LNP and thus suitable for protein expression. Although the same dose was injected from both eGFP and hIL-10 mRNAs, hIL-10 protein was expressed for a shorter time.

hIL-10 mRNA treatment proved to be effective at inducing significant morphological and functional recovery of the injured spinal cord tissue in rats. Moreover, the functional improvement was nonsignificantly greater in animals that received hIL-10 mRNA-LNPs compared to rats treated with hIL-10 protein delivered by an osmotic pump to the site of injury (Figs. 4 and 5). These data concord with findings of other laboratories on hIL-10 treatment of injured spinal cord. Park et al. [6] reported improved functional recovery after spinal cord injury in mice via virus-based IL-10 protein expression, while in another model, IL-10 treatment resulted in appearance of vast majority of anti-inflammatory type M2 macrophages in injured spinal cord [5,25]. These findings are in accordance with our results showing that tissue densities of both CD68+ macrophages and Iba-1+ microglia are downregulated by hIL-10 mRNA-LNP treatment, suggesting a strong anti-inflammatory action of hIL-10 through favorable modulation of microglia/macrophage activities. Intraspinal delivery of hIL-10 mRNA-LNP provides an excellent therapeutic approach compared with virus-based delivery methods and direct administration of hIL-10 protein through the use of osmotic pumps. mRNA-induced protein expression lacks the possible shortcomings of virus-based delivery methods and the relatively invasive nature of the implantation microsurgery of osmotic pumps [9]. In our view, delivery of IL-10 via osmotic pumps and the small diameter tubing inserted into the cavity maintained a very minor, but constant damage to the spinal cord until the pump and tubing were removed. This might have led to the minimally depressed functional improvement as compared with the intralesional delivery of hIL-10 mRNA-LNP. This fact further proves that a relatively short-term, but continuous expression of IL-10 based on mRNA delivery is at least as effective as treatment with recombinant IL-10.

Interestingly, remarkable, but short-term hIL-10 protein expression was detected in serum only on the first day after the intraspinal administration of hIL-10 mRNA-LNP, possibly due to the fact that spinal cord contusion injury leaves the blood–brain barrier open for weeks, if not months [26]. Moreover, intraspinal administration of hIL-10 mRNA-LNP is a secondary intervention after injury inducing some bleeding and macrophage/lymphocyte migration. It is also conceivable that IL-10 will also be expressed for a short time by cells entering and leaving the injured spinal cord. The very limited production of hIL-10 in the serum indicates that intraspinal mRNA delivery is a minimally invasive route of administration in experimental animals, and this procedure can likely be further improved. Proteome Profiler array investigation of the serum circulating cytokine profile resulted in only one considerable change at short term. CINC-1 showed a mild systemic decrease 2 d after administration of hIL-10 and eGFP mRNA-LNP, while the proinflammatory RANTES serum level also mildly dropped in mRNA-hIL-10 animals 5 d after hIL-10 treatment. CINC-1 is expressed by number of cells such as macrophages, neutrophils, and epithelial cells and contributes to angiogenesis as well as inflammation and wound healing [27,28]. It is thought that the mild systemic changes of these cytokines occuring few days after intraspinal hIL-10 administration are not likely to influence the repair and regenerative mechanisms in the spinal cord.

We were interested what cellular and molecular changes were induced by the hIL-10 mRNA-LNP treatment in the injured rat spinal cord. Delayed intraspinal administration of hIL-10 mRNA-LNP resulted in decreased microglial activity within the first 5 d after delivery and showed a time-delayed secondary effect with increased TIMP-1 and CNTF levels compared with the control animals. It has been shown that increased levels of circulating TIMP-1 after brain injury may contribute to the preservation of the blood–brain barrier and mediate cytoprotection via the regulation of microglial activity [29–31]. CNTF is a neurotrophic factor that promotes remyelination by grafted or endogenous oligodendrocyte precursor cells after spinal cord injury and decreases myelin loss as well as the severity of functional loss after experimental autoimmune encephalomyelitis [32,33]. PCR-based quantification of IL-6 mRNA in the spinal cord showed significantly increased levels, while TNF-a and CCL3 mRNAs decreased significantly at both examined time points in the hIL-10 mRNA-treated animals (mRNA-hIL-10) compared with the data of the mRNA-GFP group. IL-1b mRNA levels proved to be significantly decreased only on day 1 in the hIL-10 mRNA-treated animals. The limited effect of IL-10 treatment on IL-1b has also been found in other, in vitro experiments, where IL-10 in primary glial cultures (astrocytes, microglia, and mixed cell cultures) was unable to influence IL-1b levels but downregulates other cytokines, e.g., IL-6 and TNF-a [21]. While the rapid downregulation of the inflammatory cytokines IL-1b, TNF-a, and CCL3 clearly indicates the beneficial effect of hIL-10 therapy, the increase of IL-6 mRNA levels does not seem to fit into the classical view of peripheral immune responses. The increased gene expression of IL-6 can be explained by the recent findings by several, including our, publications that IL-6 may have an anti-inflammatory/immunregulatory effect in the CNS [9,34], despite its proinflammatory peripheral role. Taken together, these factors may have actively contributed to the downregulation of microglial activity and the maintenance of the integrity of spinal cord after spinal cord injury.

## Materials and Methods

### mRNA-LNP production

Codon-optimized eGFP and hIL-10 were synthesized and cloned into the mRNA production plasmid as described earlier [35]. mRNA production and LNP encapsulation were performed according to our protocols [35]. Briefly, mRNAs were transcribed to contain 101-nucleotide-long poly(A) tails. m1Ψ-5’-triphosphate (TriLink) instead of uridine-5′-triphosphate (UTP) was used to generate modified nucleoside-containing mRNA. Capping of the in vitro transcribed mRNAs was performed cotranscriptionally using the trinucleotide cap1 analog, CleanCap (TriLink). mRNA was purified by cellulose (Sigma-Aldrich) purification [36]. All mRNAs were analyzed by agarose gel electrophoresis and were stored frozen at −20 °C. Cellulose-purified m1Ψ-containing RNAs were encapsulated in LNP using a self-assembly process as previously described wherein an ethanolic lipid mixture of ionizable cationic lipid, phosphatidylcholine, cholesterol, and polyethylene glycol-lipid was rapidly mixed with an aqueous solution containing mRNA at acidic pH [37]. The ionizable cationic lipid (pKa in the range of 6.0 to 6.5, proprietary to Acuitas Therapeutics) and LNP composition are described in the patent application WO 2017/004143. The mean hydrodynamic diameter of mRNA-LNP was ~80 nm with a polydispersity index of 0.02 to 0.06 as measured by dynamic light scattering using a Zetasizer Nano ZS (Malvern Instruments Ltd, Malvern, UK) and an encapsulation efficiency of ~95% as determined using a Ribogreen assay mRNA-LNP were stored at 80 °C.

### Spinal cord injury model and intraspinal delivery of mRNA-LNP complexes into intact and injured animals

Surgical procedures and animal care were performed according to the Animal Care and Use Committee guideline at the University of Szeged. A rat contusion model of SCI was performed as described previously [9,38]. Sprague-Dawley female rats (*n* = 203; 220 to 240 g of body weight; animals were randomly chosen for the various treatment groups; for details, see Table S1) were anesthetized using ketamine hydrochloride (Ketavet, 110 mg/kg body weight) and xylazine (Rompun, 12 mg/kg body weight) and sterile precautions. After the dorsal laminectomy at the T10 vertebral level, the contusion injury was induced by using a custom-made spinal cord impactor, applying 150-kdyn force. The superficial back muscles and the skin were sutured in layers. For postoperative animal care, saline (0.9%; 5 ml) to prevent dehydration and meloxicam (Metacam; 0.5 mg/kg body weight, Boehringer Ingelheim Vetmedica) were administrated. Their bladders were manually expressed 3 times a day until reflexive function was observed. At 7 d after the injury, 3.0 μg of mRNA-LNP (eGFP-mRNA or hIL-10-mRNA) in Dulbecco’s phosphate buffered saline (PBS) were injected into animals intraspinally (3 μl) into the forming lesion cavity with a Hamilton pipette. Injured control animals (SCI group) received only contusion injury.

In intact animals, the lamina of T10 vertebra was removed and the dura was opened. mRNA-LNP (3.0 μg) was administrated into the intact spinal segment. Postoperative animal care was performed as described above. Experimental schematic of short- and long-term studies is shown in Fig. S1.

### Administration of hIL-10 via osmotic pump

One week after the contusion injury, a miniature osmotic pump (Alzet Osmotic Pumps, Cupertino, CA; type 1002, 100-μl volume, actively pumping for 2 wk) filled with hIL-10 (working concentration of 4 μg/ml, from R&D Systems, Minneapolis, MN) was placed subcutaneously in the back region. A silicone tube (Degania Silicone Ltd, Kibbutz Degania, Israel, 0.3 mm in internal diameter) extended from the pump to the spinal cord, and its distal end was inserted into the contusion cavity [9]. The tube was fixed to the surrounding musculature with 8-0 sutures (Ethilon) to avoid moving in or out of the spinal cord [9].

### Histo- and immunohistochemistry

Cross-section and longitudinal paramedian sagittal sections (25 and 16 μm in thickness) taken from the spinal cord including the lesion site were cut in a cryostat (Leica CM-1860, Leica GmbH, Germany) and mounted onto gelatinized slides. After 20 min of air-drying, the sections were permeabilized with 0.5 % Triton X-100 in PBS for 5 min and blocked for 1 h at 24 °C with 5% bovine serum albumin in PBS. Primary antibodies and lectin were used overnight at 4 °C as follows: chicken anti-GFP (1:1,000, ab13970, Abcam), rabbit anti-IL-10 (1:400, ab34843, Abcam), rabbit anti-GFAP (1:500, 7260, Abcam), goat anti-GFAP (1:500, ab53554, Abcam), rabbit anti-TUBB3 (1:500, ab18207, Abcam), mouse anti-TUBB3 (1:500, ab7751, Abcam), goat anti-Iba1 (1:400, ab5076), anti-CD68 (1:200, MAB101141, R&D Systems), and biotinylated Griffonia Simplicifolia isolectin B4 (GSA-B4, 1:200, B1205, Vector Laboratories). The following secondary antibodies were used: biotinylated goat anti-rabbit IgG (1:200, BA-1000, Vector Laboratories). The immune reaction was completed by Alexa Fluor 488 goat anti-chicken (1:600, A11039, Thermo Fisher Scientific), Alexa Fluor 488 goat anti-rabbit (1:600, A11008, Thermo Fisher Scientific), Alexa Fluor 488 donkey anti-goat (1:600, A11055, Thermo Fisher Scientific), Alexa Fluor 546 donkey anti-rabbit (1:600, A10040, Thermo Fisher Scientific), Alexa Fluor 594 donkey anti-goat (1:600, A11058, Thermo Fisher Scientific), Alexa Fluor 594 goat anti-mouse (1:600, A21203, Thermo Fisher Scientific), and Streptavidin Alexa Fluor 488 (1:600, S11223, Thermo Fisher Scientific). The sections were covered using Vectashield mounting medium containing 4′,6-diamidino-2-phenylindole (1.5 μg/ml; H-1000-10, Vector Laboratories), which labeled the nuclei of the cells. Negative controls for the secondary antibodies were performed by omitting the primary antibodies.

Immunoreactive sections were analyzed by using a BX-41 epifluorescent microscope (Olympus Ltd. Tokyo, Japan) equipped with a DP-74 digital camera and its CellSens software (V1.18, Olympus), a Pannoramic MIDI II slide scanner (3DHistech Ltd, Budapest, Hungary) equipped with Pannoramic Scanner 2.1.2 software (3DHistech), and an Olympus Fv-10i-W compact confocal microscope system (Olympus) with the Fluoview Fv10i software (V2.1, Olympus).

### Quantification of microglia/macrophage density

For quantifying CD68, Iba-1, and GSA-B4 reactivity, analysis was performed according to Arevalo-Martin et al. [39]. Four parasagittal sections (150 μm apart from each other) containing the lesion area were analyzed, 8, 9, and 12 d after injury in the SCI, mRNA-GFP, and mRNA-hIL-10 groups. Microphotographs were taken using an Olympus BX-41 epifluorescence microscope equipped with a DP-74 digital camera, and the whole spinal cord section area including the lesion area and a 2-mm-long extension of the tissue rostrally and caudally from the cavity ends was analyzed using the ImageJ software (National Institutes of Health [NIH]). The background intensity of unstained samples was individually subtracted from the intensity of treated sections. Iba-1-, CD68-, and GSA-B4-positive areas (size of areas was expressed in pixels) of the injured spinal cords were then divided by the size of examined area and multiplied by 100.

### Quantification of GFAP, TUBB3, and GSA-B4 colocalization with eGFP and hIL-10

Three spinal cords from each group were processed for immune- and histochemistry (eGFP/GFAP, eGFP/TUBB3, eGFP/GSA-B4, hIL-10/GFAP, hIL-10/TUBB3, and hIL-10/GSA-B4). The measurement was performed in a 6,500-μm-wide and 1,500-μm-high rectangle-shaped frame around the injected area. The evaluation was performed by HistoQuant software (QuantCenter multiple-module image analysis platform; 3DHISTECH). The animals that received eGFP-encoding mRNA were investigated at 6 time points (1, 2, 5, 9, 14, and 21 d) after injection, and the hIL-10 mRNA-treated rats were only examined on days 1, 2, and 5 after injection. Data were expressed as percentage of colocalized area of marker / total area of marker ratio.

### Proteome profiler arrays

The rat spinal cord fractions were homogenized in PBS with protease inhibitor (Sigma). After homogenization, Triton X-100 (Sigma) was added to a final concentration of 1%. The samples were frozen to −75 °C, thawed and centrifuged at 10,000 × g for 5 min. The supernatant was collected, and the total protein concentration was determined by using the Pierce BCA Protein Assay Kit (Thermo Fisher Scientific). The blood samples were allowed to coagulate for 1 h at room temperature and then at 4 °C overnight. The sera were collected after centrifugation of the blood at 1,000 × g for 5 min. The cytokine and chemokine contents of the samples (spinal cord and serum) were determined using the Proteome Profiler Rat Cytokine Array Kit, Panel A (R&D Systems, Minneapolis, MN). For the parallel determination of the relative levels of selected rat cytokines and chemokines, we used 390-μg total protein of spinal cord homogenates and 600-μl serum on each membrane. The spinal cord (130-μg total protein each) samples and sera (200 μl each) were pooled form 3 animals per group. The assay was performed following the manufacturer’s instructions. The chemiluminescent signals from the bound cytokines present in the spinal cord and sera were detected using the LI-COR Odyssey Imaging System and analyzed with Image Studio Software.

### RNA isolation and real-time quantitative PCR

In total, 18 rats were used for PCR analysis. Treated animals were allowed to survive 8 or 9 d after the contusion injury (1 or 2 d after mRNA treatment) in order to study the time-dependent changes in mRNA expression of inflammatory components and mediators in the spinal cord. To assess the effect of mRNA-hIL10 treatment on the expression of genes such as IL1B, IL6, TNFA, and CCL3, animals were divided into 3 groups (sham-operated, mRNA-GFP control, and mRNA-hIL10-treated). Animals in the sham-operated group were sacrificed 1 d after surgery.

Animals were transcardially perfused with physiological saline after the appropriate postoperative time, and the Th10 spinal cord segment was dissected. Spinal cord samples were homogenized in TRIzol reagent (Cat# 15596026, Thermo Fisher Scientific, Waltham, MA, USA), and the total RNA was extracted by using the Direct-zol RNA Miniprep kit (Cat#R2050, Zymo Research, Inc., Irvine, CA, USA). The amount of RNA was determined by using a spectrophotometer (Spectrostar^Nano^, BMG Labtech, Germany). To transcribe complementary DNA from RNA, Maxima First Strand cDNA Synthesis Kit was applied (Cat# K1672, Thermo Fisher Scientific). Real-time quantitative PCR was carried out using a Bio-Rad CFX96 Real-Time PCR instrument (RRID:SCR_018064; Bio-Rad, Hercules, CA, USA) and Luminaris Color HiGreen qPCR Master Mix, fluorescein (Cat# K0381, Thermo Fisher Scientific, Waltham, MA, USA) under the following parameters: 40X (95 °C/15 s, 60 °C/30 s, and 72 °C/30 s). Primer sequences for each gene were as follows: IL1B Fw: TGGCAACTGTCCCTGAACTC, Rev: AAGGGCTTGGAAGCAATCCTT; IL6 Fw: TCCGGAGAGGAGACTTCACA, Rev: GAATTGCCATTGCACAACTCTT; TNFA Fw: GATCGGTCCCAACAAGGAGG, Rev: CTTGGTGGTTTGCTACGACG; CCL3 Fw: GCTTCTCCTATGGACGGCA, Rev: CTCTTGGTCAGGAAAATGACACC. Gene expression data were normalized to glyceraldehyde-3-phosphate dehydrogenase.

### HIL-10 ELISA

The hIL-10 content of the rat samples was evaluated by using a hIL-10 ELISA kit (Sigma-Aldrich). The spinal cord samples were diluted to a total protein concentration of 2 mg/ml; the sera were diluted 2-fold, and the samples were tested in duplicate. The experiment was performed following the factory’s sandwich ELISA instructions.

Briefly, the diluted rat spinal cord and sera samples were run in 100 μl. After 2.5 h of incubation at room temperature, the plate was washed and covered with detection antibody for 1 h. The washing step was repeated, after which the Streptavidin Solution was added for 45 min. After washing the wells with 300 μl of Wash Buffer 4 times, the TMB Substrate Solution was added; then, after 30 min of incubation, the reaction was stopped by adding the Stop Solution. The absorbance was measured at 450 nm.

### Retrograde labeling and quantitative assessment of retrogradely labeled neurons

Retrograde labeling was performed as described previously [9,38]. Briefly, the L2-4 spinal segments were explored 9 weeks after the injury. At the level of L3 spinal segment, a right hemisection was performed. FB crystals (0.5 mg in each case, Chemimart GmbH, Berlin, Germany) were gently placed into the hemisection gap, and the wound was closed. Seven days after the application, the animals were reanesthetized and perfused transcardially. Transversal sections (30 μm thick) taken from the motor cortex, brainstem, and spinal cord (C2, C6, T1, and T5 spinal segments) were cut in a cryostat (Leica CM-1860, Leica GmbH, Germany) and mounted onto gelatinized slides. Every transverse section from the T5, T1, C6, and C2 spinal segments and every 5th or 10th coronal section from the brainstem or the brain were used, respectively.

### Morphometric analysis of the lesion area and spared tissue

Every fourth transverse section from the T8-L1 segments containing the lesion cavity was stained with cresyl-violet (1% aqueous cresyl-violet solution, C-1791, Sigma-Aldrich) (*n* = 4 in each group). The border between the intact tissue and the lesion cavity composed of small cysts was defined. The whole cystic cross-sectional area (lesion cavity area) at the level of the epicenter was determined as follows: the number of pixels of the reference area (1 mm^2^) and that of the cystic area was computed through the use of the NIH ImageJ analysis software (imagej.nih.gov/ij). The pixel number of the cystic area was divided by that of the reference area, and the result was expressed in percentage of lesion area compared to intact value.

The number of pixels of the spared tissue was measured at the epicenter (0) and 0.4, 0.8, 1.2, and 1.6 mm rostrally and caudally from it. Identical spinal cord segments of intact animals were used as reference values. The amount of spared tissue in the long-term groups was given as percentage of intact spinal cord values.

### Open-field test for locomotor recovery

To test the motor function recovery in the long-term survival groups (9 wk), BBB test was used at 3 d after the injury and once a week for 9 weeks [18]. Two observers evaluated the hindlimb locomotor function of animals in an open field (150 × 100 cm) for 4 min at a similar time of day for each testing.

### Video-based motor functional analysis

On the ninth postoperative week, a multiparametric kinematic analysis was carried out with a custom-made system developed in our laboratory [19,38]. The method allows the measurement of different joint angles in different moments of the step cycle. To achieve this, 2 high-speed cameras (one from lateral and one from rear aspect) and a mirror system were implemented surrounding a runway where the animals could walk into only one direction. The knee flexion, the ankle flexion, the knee lifting, and the ankle lifting parameters were recorded from lateral aspect together with the metatarsus-surface angle and tibia-surface angle observed from rear view. We chose these parameters as our earlier studies proved these to be appropriately efficient in this phase of the motor recovery [19,38].

### Statistical analysis

Student *t* test was used to compare 2 groups and 1-way ANOVA with least significant difference (LSD) post-hoc test to compare more than 2 groups. BBB scores was analyzed using repeated-measures ANOVA with LSD post-hoc test. The level of significance was set at *P* < 0.05, and all error bars represent the SEM.

## Data Availability

All data needed to evaluate the conclusions in the paper are present in the paper and/or the Supplementary Materials.

## Supplementary Materials

Supplementary Materials Table S1. The table provides information on the number of animals used for the various experimental setups and purposes. Fig. S1. Illustration depicting the short- and long-term experiments and the protocols applied. Fig. S2. eGFP expression in the intact rat spinal cord. Fig. S3. eGFP expression in Iba-1-positive cells in intact spinal cord. Fig. S4. eGFP expression in the injured rat spinal cord. Fig. S5. eGFP expression in Iba-1-positive cells in the injured rat spinal cord. Fig. S6. hIL-10 expression in Iba-1-positive cells in the injured rat spinal cord. Fig. S7. hIL-10 expression in injured rat spinal cords 2 and 5 d after intralesional administration of mRNA-LNP encoding hIL-10. Fig. S8. Decreased macrophage reaction after mRNA LNP encoding hIL-10 treatment in the injured spinal cord. Fig. S9. Decreased microglia reaction after mRNA LNP encoding hIL-10 treatment in the injured spinal cord. Fig. S10. Circulating cytokine changes in blood serum after intralesional administration of mRNA-LNP.
