# Supplementary material for: Restoration of Motor Function through Delayed Intraspinal Delivery of Human IL-10-Encoding Nucleoside-Modified mRNA after Spinal Cord Injury
Source: Research (Wash D C). 2023 Mar 9;6:0056. doi: 10.34133/research.0056 (PMC10013810; doi:10.34133/research.0056)
Supplement: Supplementary Materials — Table S1. The table provides information on the number of animals used for the various experimental setups and purposes. Fig. S1. Illustration depicting the short- and long-term experiments and the protocols applied. Fig. S2. eGFP expression in the intact rat spinal cord. Fig. S3. eGFP expression in Iba-1-positive cells in intact spinal cord. Fig. S4. eGFP expression in the injured rat spinal cord. Fig. S5. eGFP expression in Iba-1-positive cells in the injured rat spinal cord. Fig. S6. hIL-10 expression in Iba-1-positive cells in the injured rat spinal cord. Fig. S7. hIL-10 expression in injured rat spinal cords 2 and 5 d after intralesional administration of mRNA-LNP encoding hIL-10. Fig. S8. Decreased macrophage reaction after mRNA LNP encoding hIL-10 treatment in the injured spinal cord. Fig. S9. Decreased microglia reaction after mRNA LNP encoding hIL-10 treatment in the injured spinal cord. Fig. S10. Circulating cytokine changes in blood serum after intralesional administration of mRNA-LNP. [file research.0056.f1.docx]

**Supplementary Materials**

**Supplementary Table 1. The table provides information on the number of animals used for the various experimental setups and purposes.**

| Exp. groups  Procedures | Intact | SCI | mRNA-GFP | mRNA-hIL-10 | osm-hIL-10 | sham |
| --- | --- | --- | --- | --- | --- | --- |
| immunohistochemistry for short term study | 18 | 18 | 36 | 9 | - | - |
| ELISA | - | 12 | 12 | 12 | - | - |
| Proteome profiler | - | 12 | 12 | 12 | - | - |
| PCR | - | - | 6 | 6 | - | 6 |
| morphological analysis, Fast Blue labelling and functional tests for long term study | - | 8 | 8 | 8 | 8 | - |


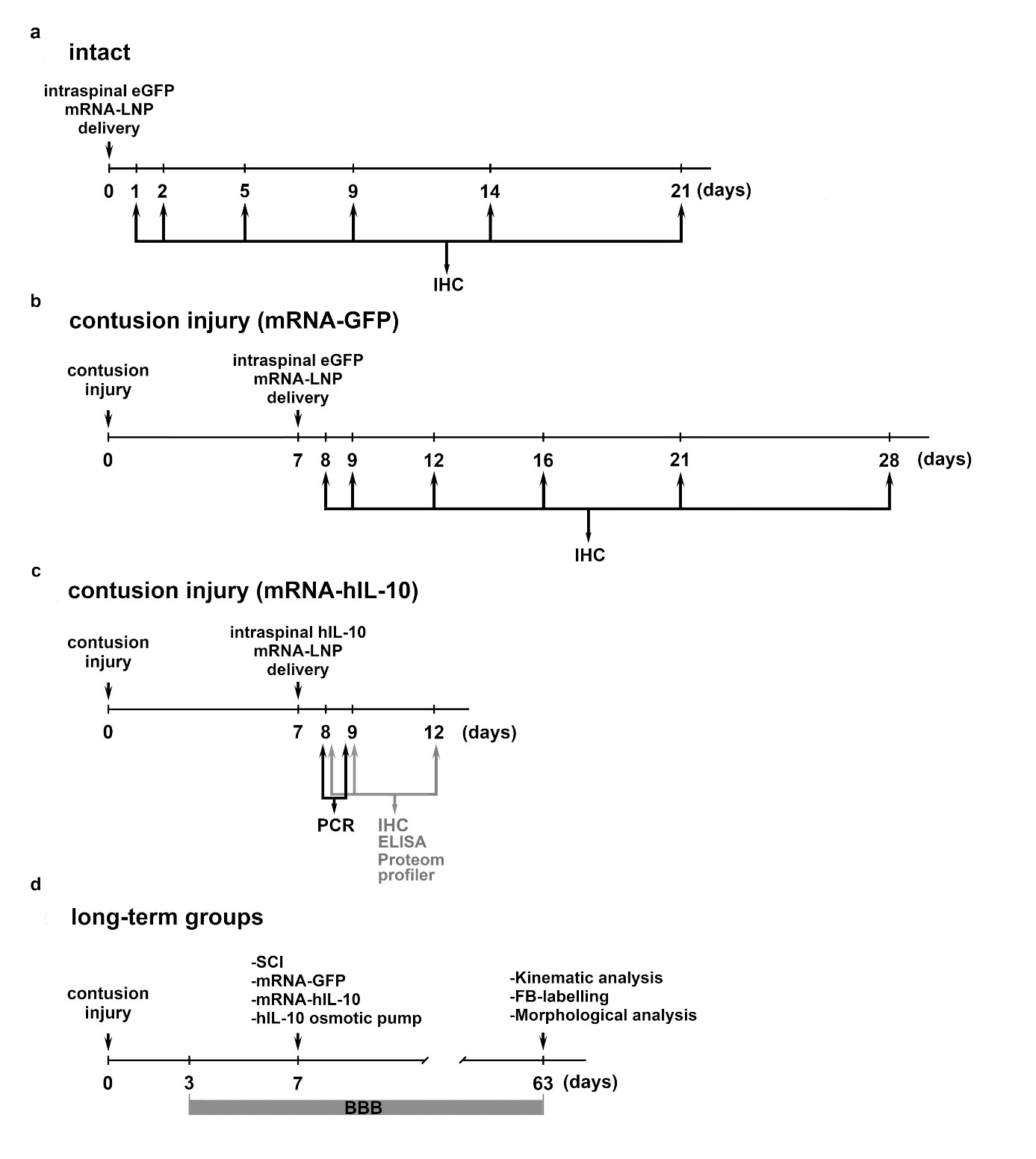


**Supplementary Figure 1. Illustration depicting the short- and long-term experiments and the protocols applied.** Intact adult female rats (220 to 250 g) underwent contusion injury at the T10 vertebral level. On day 7 after the injury, a single dose of mRNA-LNP (3.0 μg) or saline was administered in to the injured rat spinal cord. **a-c)** In the short-term study at 1, 2, 5, 9, 14 and 21 days after the injection, the spinal cords were collected from the intact (**a**) and injured rats (**b**) that received saline or mRNA-LNP encoding eGFP. Immunohistochemistry (IHC) was performed to study the eGFP expression at various time points. **c)** Another group of rats were treated with mRNA-LNP-encoding hIL-10 1 week after the injury. The hIL-10 expression was analyzed 1, 2, 5 and 9 days after mRNA-LNP treatment. Other rats were used for various assays to analyze the acute inflammatory response and hIL-10 expression. **d)** In the long-term study, open field locomotor test (BBB) was applied on day 3 after SCI, followed by weekly assessments up to 9 weeks in the following groups (SCI, mRNA-GFP, mRNA-hIL-10 and osm-hIL-10 group). Gait parameters were determined through the use of a plexiglass runway equipped with a mirror system and cameras recording both lateral and rear-view aspects. Retrograde labelling from the L3 spinal segment and morphological analysis were performed to identify the number the retrogradely labelled neurons rostral to the injury and quantify the tissue preservation at the lesion site, respectively.


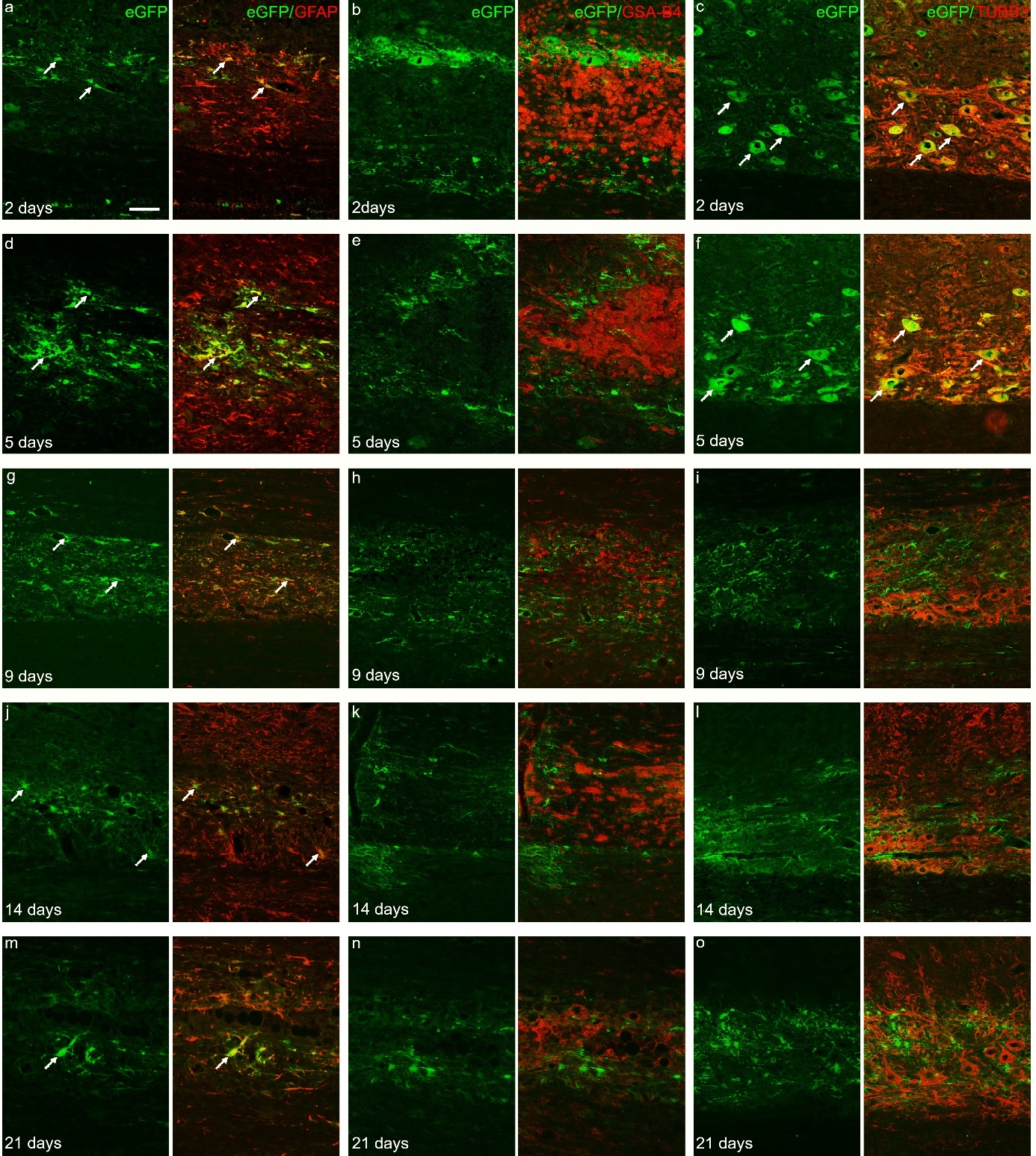


**Supplementary Figure 2. eGFP expression in the intact rat spinal cord.** **a-c)** Rat astrocytes and neurons but not GSA-B4 cells show strong eGFP expression at 2 days post-injection**. d-f)** Similar eGFP expression can be seen 5 days after delivery of mRNA-LNP. **g-i)** Nine days after mRNA-LNP administration only astrocytes express eGFP. **j-o)** Astrocytes expressed eGFP up to 21 days after intraspinal mRNA-LNP administration. Arrows show co-localizing cells. Scale bar in **a=**50 µm.


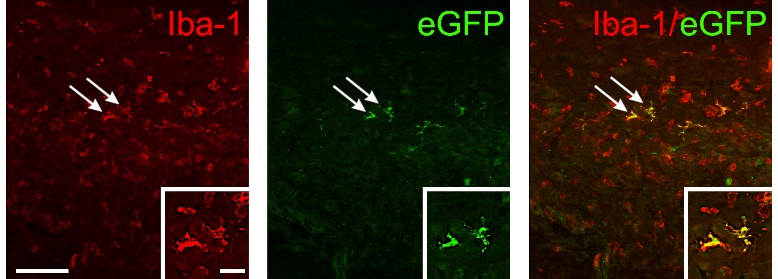


**Supplementary Figure 3. eGFP expression in Iba-1 positive cells in intact spinal cord.** Representative images show Iba-1-positive cells co-localized with eGFP in the intact spinal cord 1 day after intraspinal delivery of mRNA-LNP encoding eGFP. Arrows indicate the co-localized cells. Scale bar: 100 µm and 20 µm


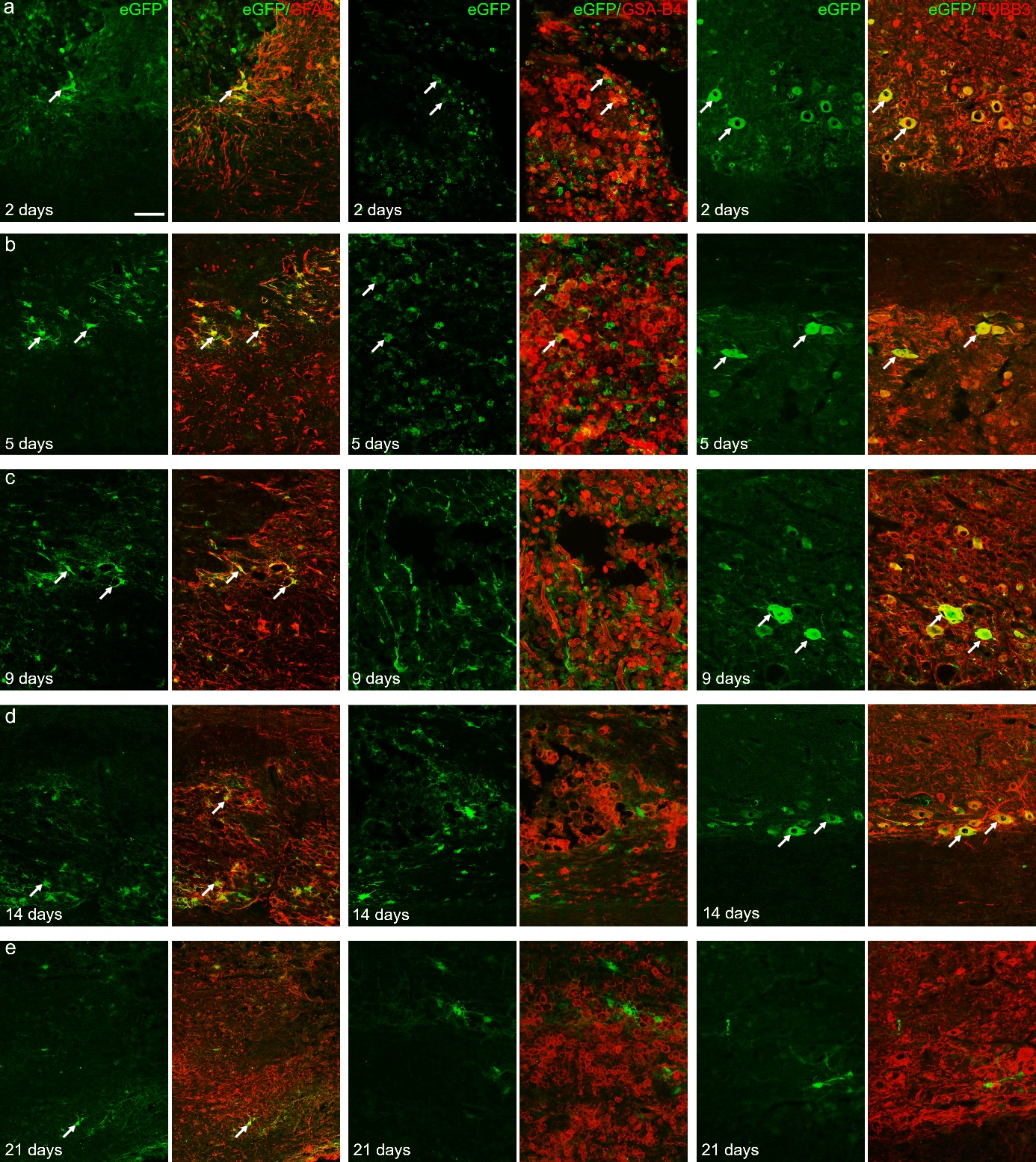
 **Supplementary Figure 4. eGFP expression in the injured rat spinal cord. a, b)** Astrocytes, (GFAP), microglia/macrophages (GSA-B4) and neurons (TUBB3) displayed strong eGFP expression 2 and 5 days after mRNA-LNP delivery in or around the lesion area. **c, d)** Images demonstrate the presence of eGFP in astrocytes and neurons, but not in GSA-B4-positive cells 9 and 14 days after mRNA-LNP delivery. **e)** On day 21 after intraspinal mRNA-LNP treatment only astrocytes co-localized GFAP with eGFP. Arrows show co-localizing cells. Scale bar in **a=**50 µm.


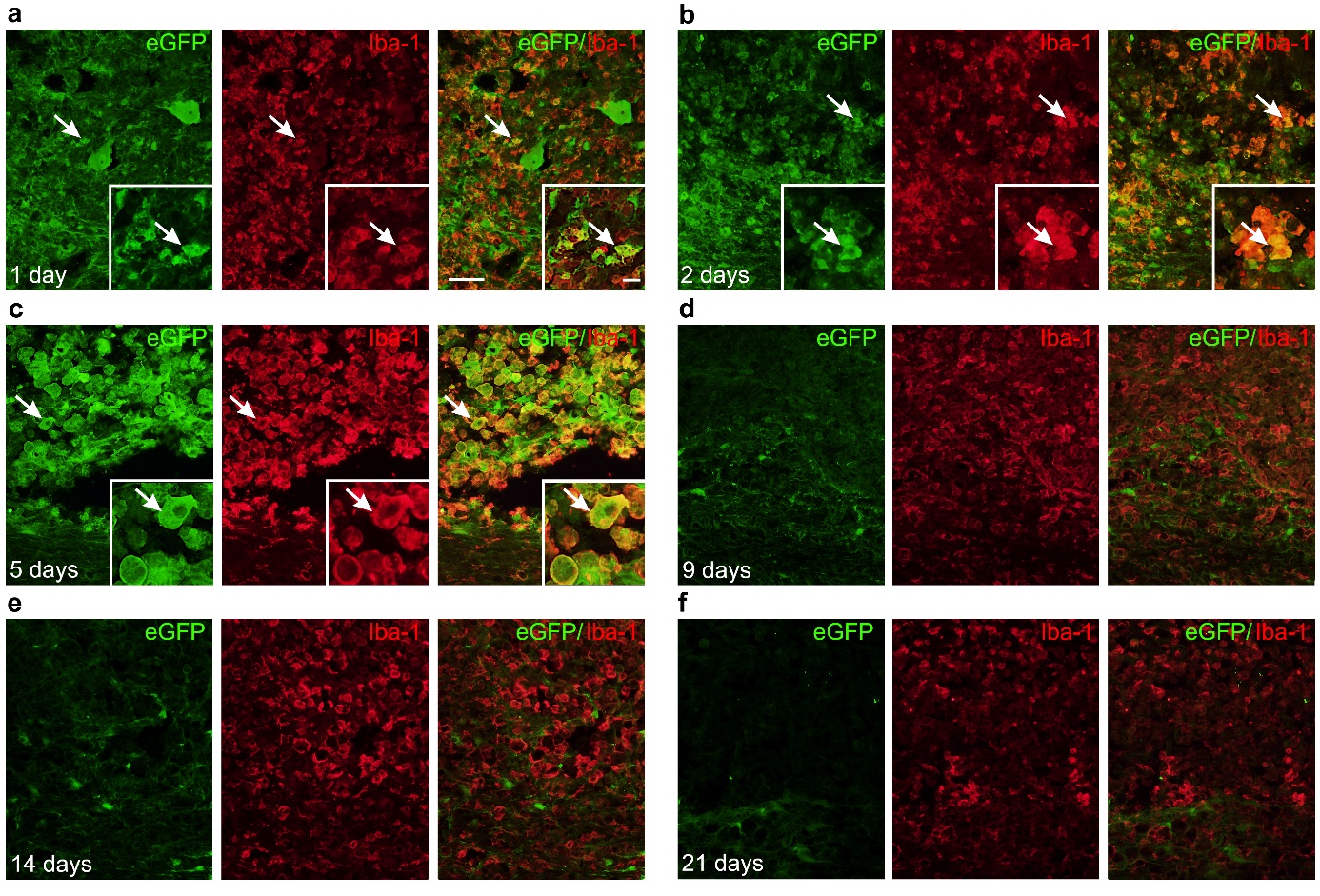


**Supplementary Figure 5. eGFP expression in Iba-1-positive cells in the injured rat spinal cord. a-c)** Images demonstrate the presence of eGFP in Iba-1-positive cells 1, 2 and 5 days after mRNA-LNP delivery in or around the lesion area. **d-f)** On days 9, 14 and 21 after intraspinal mRNA-LNP treatment no eGFP-positive microglia cells were detected. Arrows show co-localized cells. Scale bar in **a=**50 µm and 20 µm.


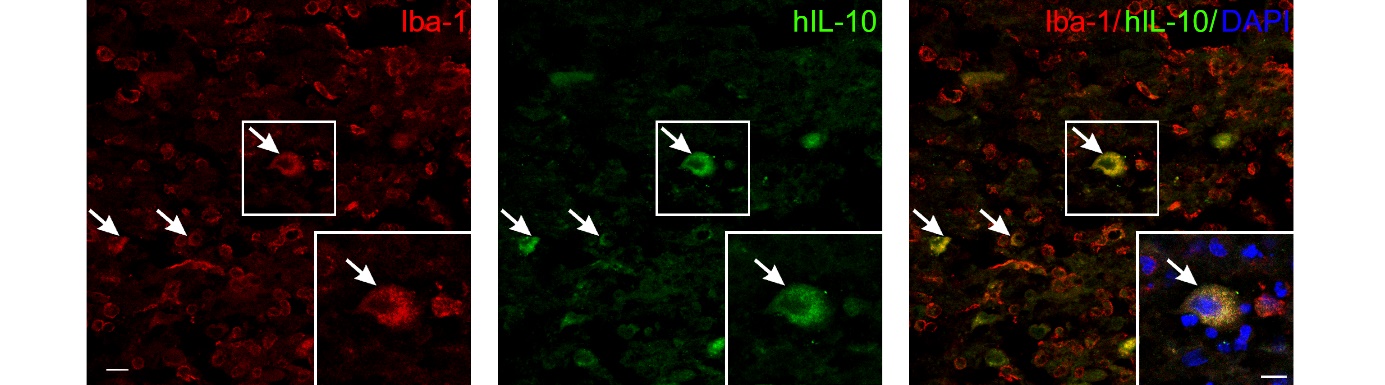


**Supplementary Figure 6. hIL-10 expression in Iba-1-positive cells in the injured rat spinal cord.** Images show Iba-1-positive cells co-localized with hIL-10 in injured rat spinal cord 1 day after intralesional delivery of mRNA-LNP encoding hIL-10. Scale bar: 20 µm


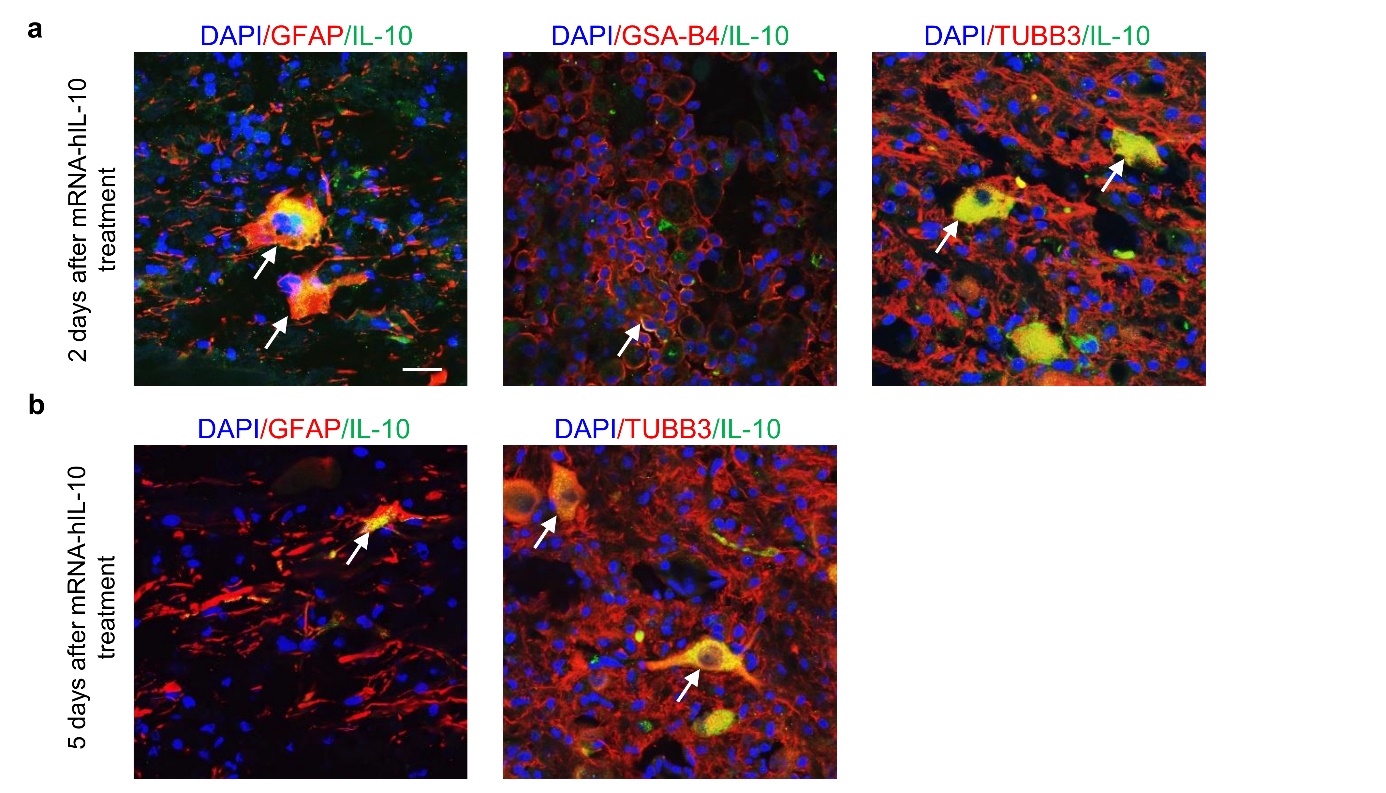


**Supplementary Figure 7. hIL-10 expression in injured rat spinal cords 2 and 5 days after intralesional administration of mRNA-LNP encoding hIL-10. a)** Confocal images show hIL-10 expression 2 days after intraspinal LNP administration in astrocytes, in GSA-B4-positive cells and in neurons. **b)** Five days after the intraspinal LNP delivery hIL-10-positive neurons and astrocytes are present in the rat spinal cord. Arrows show co-localizing cells. Scale bar in **a** 25 µm.


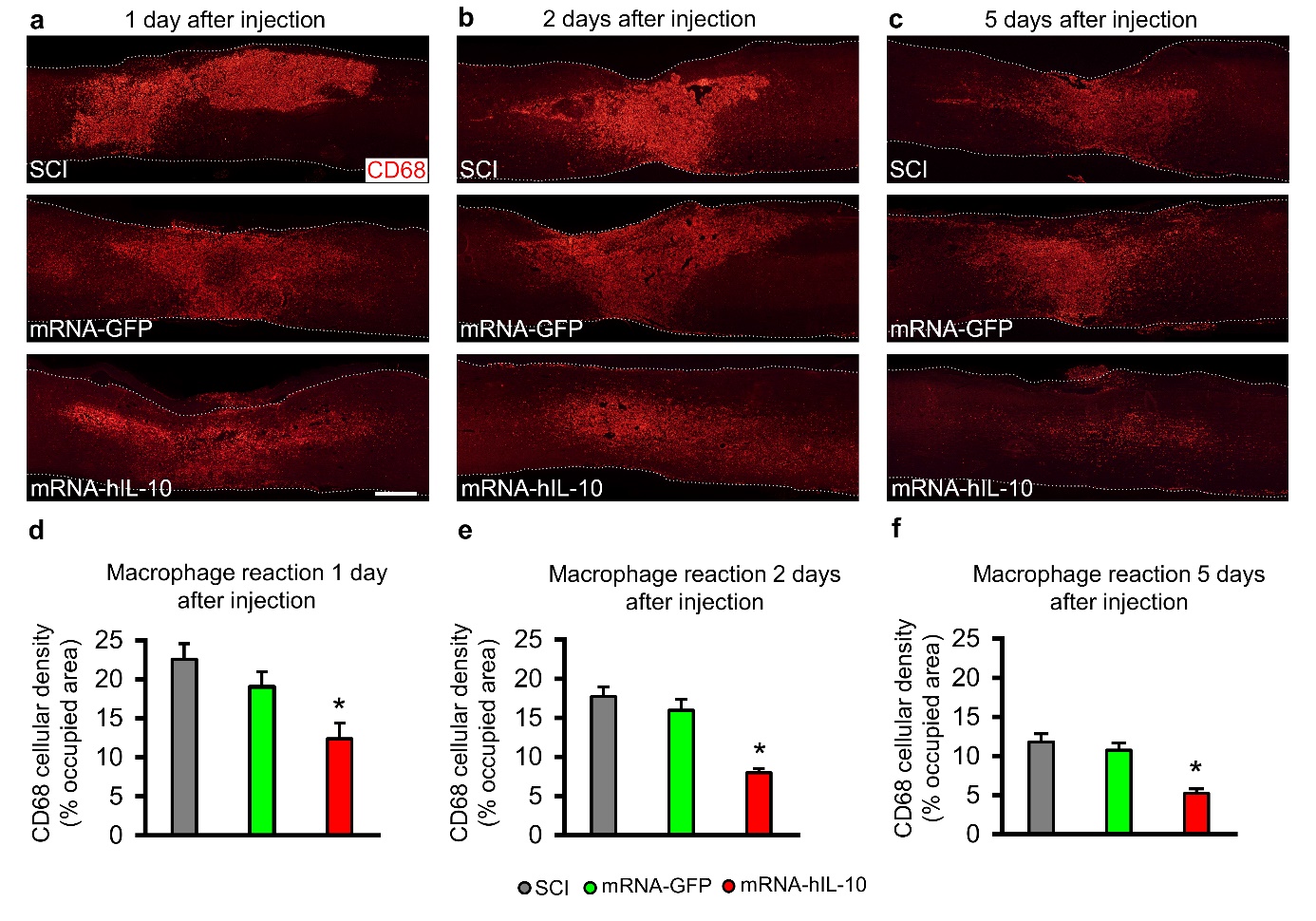


**Supplementary Figure 8. Decreased macrophage reaction after mRNA LNP encoding hIL-10 treatment in the injured spinal cord. a-c)** Representative images of paramedian sagittal spinal cord sections show CD68-posititev cells 1, 2 and 5 days after intralesional delivery of saline (**a**), mRNA-LNP encoding eGFP (**b**) and mRNA-LNP encoding hIL-10 (**c**) in the injured spinal cord. **d-f)** Quantification of CD68 density in the sagittal sections of the spinal cord revealed significantly decreased level of CD68 at all examined time points in the hIL-10 mRNA treated group (mRNA-hIL-10) compared with the SCI and mRNA-GFP groups. Data were analysed by using one-way ANOVA with LSD multiple comparisons test. Data represent the mean ± S.E.M. **d, e** and **f**) n=4, biologically independent experiments. **p* < 0.05 indicates significant difference between SCI, mRNA-GFP vs. mRNA-hIL-10. Scale bar in **a**=300 µm.


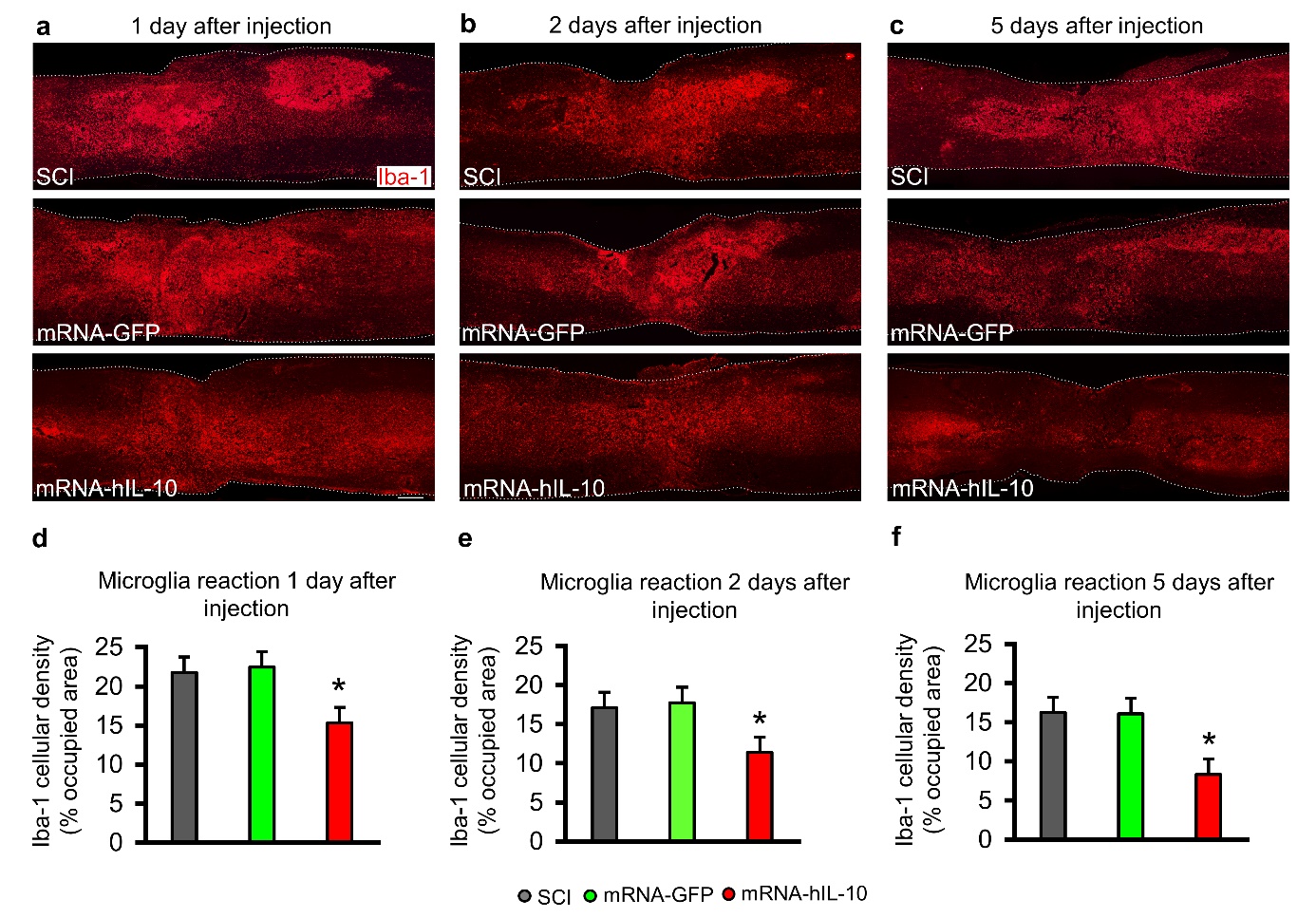


**Supplementary Figure 9. Decreased microglia reaction after mRNA LNP encoding hIL-10 treatment in the injured spinal cord. a-c)** Representative images of paramedian sagittal spinal cord sections show Iba-1-positive cells 1, 2 and 5 days after intralesional delivery of saline (**a**), mRNA-LNP encoding eGFP (**b**) and mRNA-LNP encoding hIL-10 (**c**) in the injured spinal cord. **d-f)** Quantification of Iba-1 density in the sagittal sections of the spinal cord revealed significantly decreased level of Iba-1 expression at all examined time points in the hIL-10 mRNA treated group (mRNA-hIL-10) compared with the SCI and mRNA-GFP groups. Data were analysed by using one-way ANOVA with LSD multiple comparisons test. Data represent the mean ± S.E.M. **d, e** and **f**) n=4, biologically independent experiments. **p* < 0.05 indicates significant difference between SCI, mRNA-GFP vs. mRNA-hIL-10. Scale bar in **a**=300 µm.


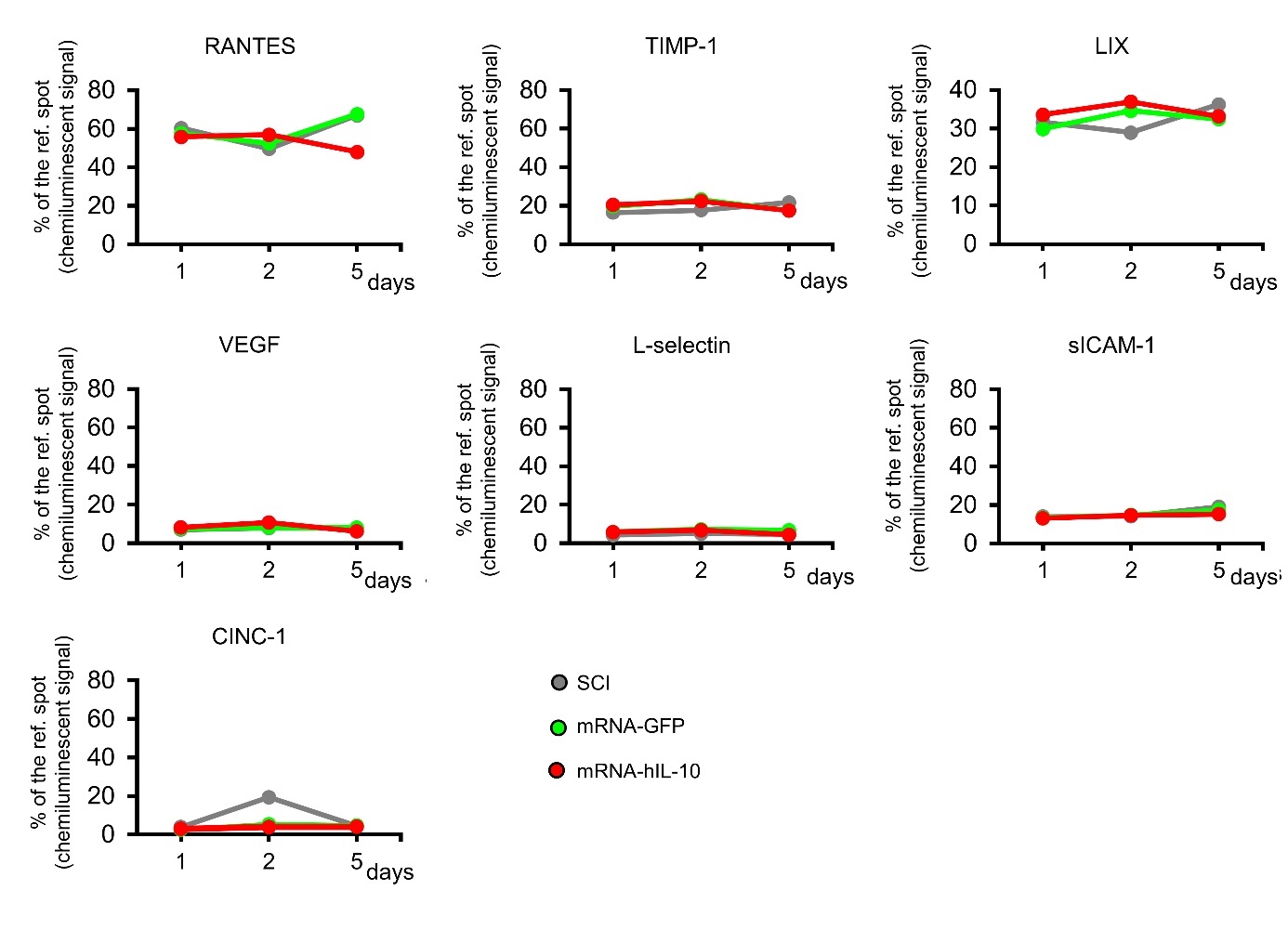
**Supplementary Figure 10. Circulating cytokine changes in blood serum after intralesional administration of mRNA-LNP.** Rat cytokine changes were assessed with the Proteome Profiler array, which compares the relative levels of 29 cytokines. The chemiluminescent signals of spots were compared with reference spots and expressed as % of the reference spot. Abbreviations: RANTES, Regulated upon Activation, Normal T Cell Expressed and Presumably Secreted; TIMP-1, Tissue inhibitor of matrix metalloproteinase 1; LIX, Chemokine (C-X-C motif) ligand 5 (CXCL5); VEGF, Vascular endothelial growth factor; L-selectin, CD62L (a cell adhesion molecule); sICAM-1, Soluble intercellular adhesion molecule-1; CINC-1, Cytokine-induced neutrophil chemoattractant 1.
